# Putting FUN into involvement: feedback user needs in the design of a mobile phone app for people with long-term conditions

**DOI:** 10.1186/s40900-026-00837-0

**Published:** 2026-01-24

**Authors:** David C. Clayton, Michelle Hadjiconstantinou, André G. NG, Pamela Andrade, Jo Bell, Fola-Dami Eyitemi, Rachel Hobson, Asad Masood, Marios Panagi, Asad Raza, Eda Tonga, Hannah Worboys, P-STEP User Group, Umesh Kadam

**Affiliations:** 1 Cardiovascular Sciences, School of Medical Sciences, University of Leicester, University Road, Leicester, LE1 7RH UK; 2 Division of Global, Diabetes Research Centre, Lifestyle and Metabolic Health, College of Life Sciences, University of Leicester, Leicester, UK; 3 Leicester Diabetes Centre, Leicester General Hospital, Leicester, UK; 4 NIHR Leicester Biomedical Research Centre, University of Leicester, Leicester, UK; 5 BHF Leicester Centre of Research Excellence, NIHR Leicester Biomedical Research Centre, University of Leicester, University Road, Leicester, LE1 7RH UK; 6 NIHR Leicester Biomedical Research Centre, Leicester General Hospital, Leicester, LE5 4PW UK; 7 School of Computing and Mathematical Sciences, University of Leicester, University Road, Leicester, LE1 7RH UK; 8 School of Medical Sciences, Respiratory Sciences, University of Leicester, University Road, Leicester, LE1 7RH UK; 9 Climate and Atmosphere Research Centre, The Cyprus Institute, Nicosia, 2121 Cyprus; 10 Department of Global, Lifestyle and Metabolic Health, School of Medical Sciences, Diabetes Research Centre, University of Leicester, University Road, Leicester, LE1 7RH UK; 11 Biostatistics Research Group, Public Health and Epidemiology, School of Medical Sciences, University of Leicester, University Road, Leicester, LE1 7RH UK; 12 Department of Health & Community Sciences, University of Exeter, St Luke’s Campus, Heavitree Road, Exeter, EX1 2LP UK

**Keywords:** PPI involvement, Engagement, Long-term conditions, Older people, Design, Digital, Mobile phone app, mHealth, Exercise, Climate, Environment, Evaluation, User experience

## Abstract

Involving users in the design of health mobile phone apps is important to their success. This article reports on the involvement activities of people with long-term health conditions in a project called P-STEP. This is a unique project combining health and environmental data within a mobile phone app to offer advice to people with long-term conditions on walking time and where to walk to avoid air pollution. The article aims to highlight a locally created involvement approach called FUN (Feedback User Needs) and to showcase the difference this made both to the end product (the mobile phone app prototype) and to the user’s experience (how users felt about being involved in the project). Firstly, by focusing on the discussion groups and validation workshops, the article will show how users made a difference and had an impact on the design. Secondly, users contributed to the writing of this article and provided written narratives of their experiences. These experiences are used to reflect on the quality and consistency of our approach with users and what made a positive difference to them in being involved. Overall, the FUN approach was successful in ensuring that user contributions influenced the design of the app and provided a positive experience of the project. We offer this approach as an example of involving users in the design of a health mobile phone app and the methods for encouraging positive involvement outcomes in similar projects.

**Supplementary Information:**

The online version contains supplementary material available at 10.1186/s40900-026-00837-0.

## Abstract (plain language summary)

This article is about how people with long-term conditions were involved in the design of a mobile phone app. Mobile phone apps can fail because they do not meet people’s needs. Involving people who may use the app is therefore very important. In this article, we write about how we involved potential users and what difference they made to the design. We also asked for the views of users to include in the article. We called our approach FUN, which stands for Feedback User Needs, because it was about feeding back what users needed to the different teams who created the app. Our approach included online discussion groups and face-to-face workshops that brought users and design staff together, and written documents to show the designers what users said. We found that using FUN enabled users to influence the design of the app and feel they made a difference. We wanted to share our work with others as a good example of involving people with long-term conditions in the design of a mobile phone app.

**Supplementary Information:**

The online version contains supplementary material available at 10.1186/s40900-026-00837-0.

## Background

This article reports on the involvement of people with lived experiences of long-term conditions in a project called in P-STEP^1^ (Personalised Space Technology Exercise Platform). The long-term conditions supported by P-STEP include heart disease, lung disease and type 2 diabetes. P-STEP is a unique project, funded by the European Space Agency and the University of Leicester, combining health and environmental data within a mobile phone app to offer advice to people with long-term conditions on walking time and where to walk to avoid air pollution. The app aims to enable users to optimise the benefits of walking without incurring negative impacts due to factors such as air pollution.

### Exercise and long-term conditions

According to the World Health Organisation (WHO), mortality from conditions like cardiovascular disease, stroke, and diabetes can be prevented through lifestyle changes [1]. Exercise is important to health in general but particularly for those with long-term conditions. Although regular exercise can help prevent and treat long-term conditions, many with those conditions remain inactive [2]. It is recommended that at least 150 min of moderate-intensity aerobic activity per week be undertaken by the UK chief medical officer [3]. However, many people do not meet these guidelines for reasons such as finding the time and being motivated to exercise [4]. As a P-STEP user stated …

*More to the point*,* perhaps – those with LTC may feel that this target is unattainable*,* and are therefore demotivated. They may also experience fluctuations in their health*,* so this needs to be factored into the programme. [P-STEP User reviewing this article]*

### Air pollution

Air pollution can impact respiratory and heart conditions, including increased risk for asthma, heart attacks, stroke, and premature mortality. Studies have found evidence that short-term exposure to air pollution could have negative effects on health [5–9].

### Use of mHealth technology

mHealth, including mobile phone apps, has the potential to help motivate people with long-term conditions to exercise and avoid the negative environmental conditions that may impact their conditions, like air pollution [10]. Such applications are becoming widespread as they offer an affordable way of reaching people who may benefit from this type of health intervention [11]. Mobile phone apps are increasingly being used to support people to manage their conditions, tracking symptoms, providing therapeutic advice, and offering self-management messages to enable behavioural change [1]. However, poor experiences using an app can lead to failure if the design fails to meet the needs of the targeted users [12]. It is estimated that engagement of 75% of mobile phone apps drops within the first few weeks of registration [11]. Reasons for this may be due to technical difficulties experienced by the user, lack of features to support the user throughout their app journey, or lack of perception of the usefulness and value of the app [13].

### User involvement and mobile app design

Involving people with lived experience of long-term conditions plays a key role in developing suitable health programmes like mobile phone apps [14], and their perspectives can act as a key factor in successful uptake and engagement of health programmes [15]. Mobile phone app design employs the concept of an ‘end user’ and UX (User eXperience) [16]. User Centred Design (UCD) is an approach to software development that aims to make systems usable and useful by focusing on the users’ needs and requirements. The aim is to enhance effectiveness and efficiency, human well-being, user satisfaction, accessibility and sustainability, and counteract possible negative effects of use on people’s health, safety, or performance [17].

In supporting User Centre Design, users are not typically ‘research’ subjects but seen as collaborators being consulted at every stage of the design process to help make decisions and test and validate the implementation of an app. As Davies and Mueller (2021) [1] argue, there is a difference between conducting ‘research’ on people and ‘involving’ people in the design of technology. With research, the characteristics and behaviour of people are studied as research subjects, whereas in User Centred Design, users take an active role and contribute to the study or product/service design. Parallels exist with Public and Patient Involvement and User-Centred Design approaches, where the activities support ‘doing with’ rather than ‘to’ patients and/or members of the public [18]. Distinguishing these activities as involvement or research can be difficult [19] but for this project, user involvement was about influencing decisions on the design of the app and not collecting data on them. The term ‘user’ is used throughout this article to indicate those people with lived experiences of long-term conditions who contributed to and could influence the design of P-STEP.

### Involving users in P-STEP

A challenge for P-STEP was to make the app acceptable and usable for the users for whom it was intended. Accordingly, it was paramount to involve people with lived experience from the outset to ensure the design is acceptable and usable for their intended participants/users. The intention was for a high level of involvement of these users in the design of P-STEP to ensure the early prototype successfully met user needs and requirements.

Despite the proliferation of mobile phone apps, there is little evidence of the best way to do this involvement [20]. A recent systematic review on the involvement of older people in technological design, for example, found that people were likely to be involved in projects periodically being surveyed or observed using the technology rather than contributing in a more ‘democratic’ way [21]. Platt [22] has also suggested that designers may focus on the ‘technology’ and software rather than on developing the preferences of users that would make a difference in using them. Arguably, there is a lack of understanding of the importance of involvement in this respect, where the influence of users on the design of apps may be included or ignored at the discretion of the designers [21].

### P-STEP and the FUN approach

For P-STEP, involvement was conceived from a very early stage and was integral to the app’s development. As one user described this… *“a ‘bottom-up’ approach*,* rather than ‘top down’ involvement”*. The project followed a person-centred approach [23] to involving users called Feedback Users Needs (FUN). A primary aim of FUN was to collaborate and develop mutual relationships between users and the teams involved in P-STEP. Similar to User-Centred Design, which focuses on optimising usability and user experience through iterative testing and co-design [24], FUN aimed to actively involve users in the design process to foster shared ownership. However, in contrast to User Centred Design, FUN attempted to be more participatory and democratic, emphasising trust and relationship building between all those involved by drawing on values such as empowerment and treating people with dignity and respect. FUN was about creating constructive conversations; bringing together the users and the teams to work together on an equal footing and bring their respective experiences to provide relevant solutions in the design of the app.

This approach was influenced by the UK Standards for Public Involvement [25] and National Institute for Health and Care Research (NIHR) guidance on involvement [18]. The UK Standards are designed to improve the quality and consistency of public involvement in research but can be used to support public involvement across other domains [26]. As a framework for what good public involvement looks like, the standards include 6 domains - inclusive opportunities, working together, support and learning, governance and communication. In line with this standard, FUN sought to create a welcoming, safe, and inclusive space for users, respecting all views and using inclusive methods which would reflect the different needs of users involved in the P-STEP project.

### Aim of this article and involvement questions

This article aims to describe the FUN approach and consider what impact it made both to the design of P-STEP and the user’s experience (how the experience of being involved affected/impacted them). We will also consider the question of what good involvement looks like in the context of this digital health project and what factors make for good involvement that may be transferable to other contexts and areas of involving public/patient involvement.

The development of health apps will involve gathering intelligence from different sources to inform the design. In addition to involving people with lived experience of long-term conditions, we also involved Healthcare professionals (HCPs) stakeholders. However, for this article, we will only describe the involvement of people with long-term conditions, whom we describe as the ‘core’ user group.

## Methods

### Operation of user involvement in P-STEP

As a multi-disciplinary and multi-factorial project, designing the building P-STEP required a high level of organisation and coordination. Several teams were involved, including a health, environmental, app build, and user involvement team, each consisting of a team leader, research associate and subject experts. These teams were managed by a Project Management Team, consisting of a project manager, project coordinator and project lead. To create relationships and support the development activities performed by the app developers^2^, it was agreed to have regular contact with users (every three weeks) to fit with the iteration cycle and enable opportunities for the teams to interact with users regularly. To ensure regular contact, a ‘core’ user group was established.

### Invite to the ‘core’ user group

An invitation to join the user group followed a previous engagement session run by the University Hospitals of Leicester Trust. An advertisement for volunteers was sent by the Trust in April 2022 to previous patients already involved in their public and patient groups. The invitation to the group was purposeful in aiming to recruit a diversity of people with long-term conditions (heart, respiratory, and diabetes diseases). Twelve people volunteered in the first instance. Due to dropouts, the number was reduced to ten. These ten users became the P-STEP user group (colloquially called the ‘psteppers’). The ten users included eight women and two men, who had self-reported long-term conditions as specified in the original advertisement, including asthma, chronic obstructive pulmonary disease (COPD), heart disease, and type 2 diabetes. This included users who had more than one condition, such as COPD and a heart condition. Three users were drawn from the South Asian community, the others from the White British community. The ages of the group were between 50 and 80.

### User involvement phases and methods of user engagement

User involvement was categorised into two phases: (i) design; (ii) validation (see below). The design phase was based on undertaking a series of online discussion groups with users, and the validation phase on running two interactive workshops (see below). In line with our FUN approach, we adopted a pragmatic approach to facilitating discussions with users. In part, this was to ensure their views could be passed in a timely way to the app-building team to support the development, given the project timelines (a three-weekly iteration cycle). Although meetings were recorded, they were not fully transcribed; rather, recordings were used to identify the key points and/or themes from the discussions with respect to the specific topic considered [19]. The overall aim is to shape and guide the design of the app and not to research the users. A variety of methods were utilised in the activities to help users influence the teams and provide feedback to them on how their views were making a difference to the design. These included:

- Presenting visualisations from the app for discussion
- Storytelling, presenting case studies and vignettes for discussion
- Structured and non-structured questions about the designs
- Quick-fire voting
- Observations during testing of the prototypes
- Users’ booklets for them to record interaction with the app

The workshops also utilised some adapted research tools to help users provide feedback to the teams. The System Usability Scale (SUS) questionnaire [27] is a questionnaire used to assess the usability of a product or system. This consisted of 10 questions with 5-point Likert scale responses ranging from “Strongly Disagree” to “Strongly Agree”. This was adapted for use with the workshops (see Additional File 3, page 4). The (P-STEP) User Engagement Scale – Short Form (UES-SF) [28] is based on a questionnaire designed to capture key aspects of user engagement - Focused Attention, Perceived Usability, Aesthetic Appeal, and Reward (see Additional File 3, page 4). These were tools familiar to the app development team, and coupled with discussion from users about how they scored, supported useful conversations about the app design (see results section below).

The period of involvement in the design of the app was from May 2022 until April 2023. The planning of discussions and activities was a collaboration between the different teams. Topics were identified by different teams at project meetings. These topics would then be discussed with users and their feedback provided back to the designers to inform future topics. One Research Associate coordinated the involvement work and facilitated the discussion groups, and another undertook visual design, translating topics and user responses into wireframes for the app team to build. The user planning involved two academic experts, the Project Manager and the Project Administrator, who supported the planning and running of sessions. Research Associates for health and environment, respectively, would take part in discussion groups based on their particular topics and provide feedback to their teams.

#### The design phase and user group discussions

The design phase involved discussion with users to identify their needs to inform the system requirements. During this time, there were uncertainties about the COVID-19 pandemic, and given the risk to users with long-term conditions (as was the target group of users for P-STEP), discussion groups were undertaken online. The discussion groups were run for about one hour and undertaken approximately every three weeks, with the attendance of members of the different teams on their relevant topics. Table 1 highlights the number of users and research associates attending the discussion groups. Further highlights of all the discussion groups, topics, and their outcomes are provided in Additional File 1. The user’s time to attend sessions was reimbursed using ‘thank you’ payments based on NIHR guidance [29].

**Table 1:** Attendees of the discussion groups

Discussion groups and topics	Users attending	Design Team attending	Total attendees
1. Introductions	10	3	13
2. Health Recommendations	7	4	11
3. Walking and air quality information	9	4	13
4. Motivation	6	2	8
5. Visualisations of P-STEP	10	4	14
6. Messages and notifications	9	4	13
7. Setting goals and home screen	9	3	12
8. Messages and visualisations update	10	5	15
9. Home screen, routes and website	9	3	12
10. Reflections on the app and Christmas	9	2	11

Values and ground rules were shown at the beginning of each session (see Box 1), along with updates and feedback from previous sessions. With the permission of the group, the online discussions were recorded and key messages communicated in the writing of a ‘FUN Document’ after each session (see Additional File 2 for an example). The FUN document drew together the discussions and highlighted any consensus and differences in views from users. The document was uploaded onto a shared platform, allowing opportunities for the teams to comment and raise questions about the discussions and agree on the content. This would then inform the visualisations of the app and what was to be implemented in the design by the app build team.

**Box 1:** P-STEP values

• Treating others with dignity and respect
• Treating people as individuals - their culture, lifestyle, unique personal history, likes and dislikes
• Seeing things from different perspectives
• Valuing everyone’s contribution and knowledge

#### The validation phase

**Fig. 1:**
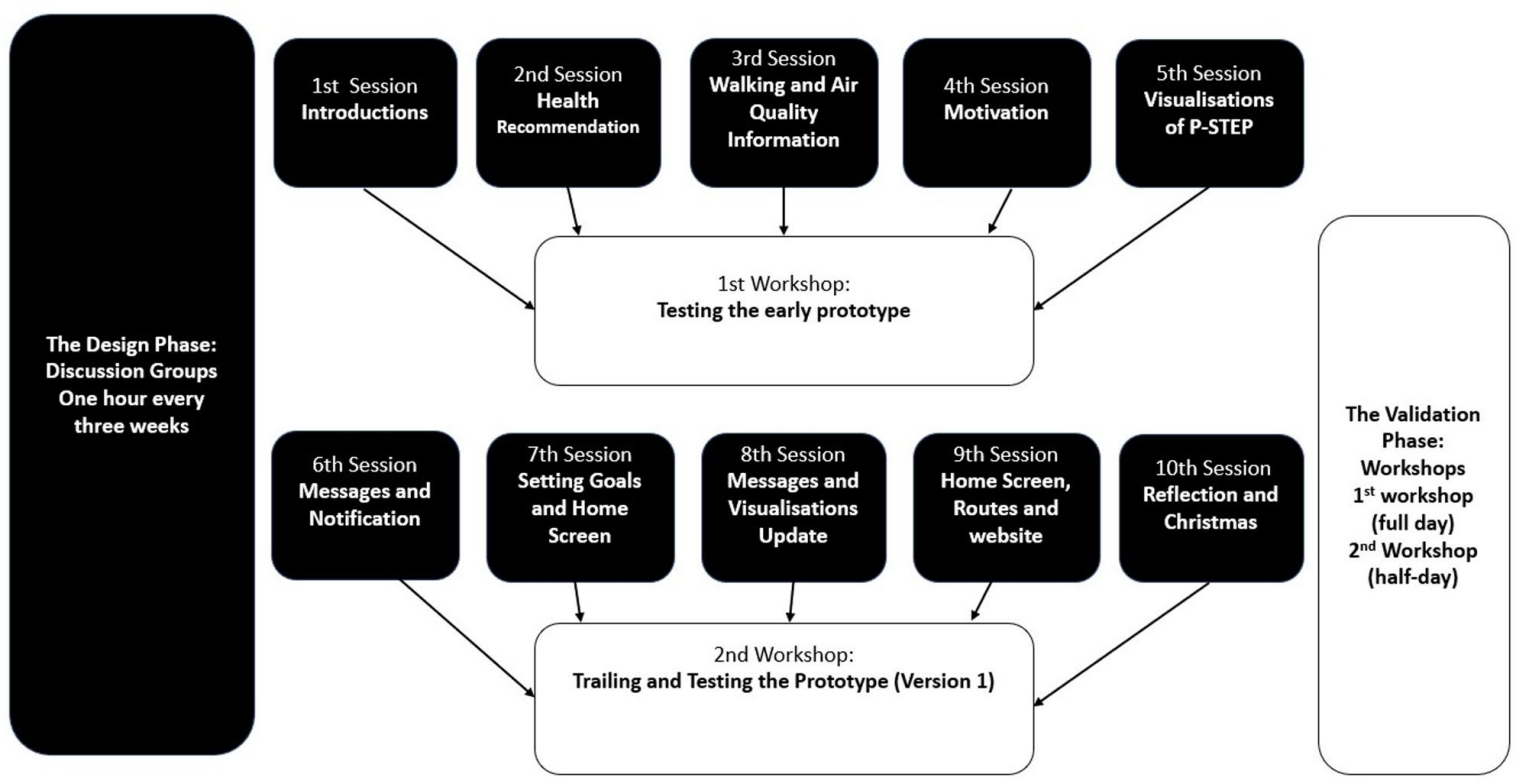
User involvement session in P-STEP

Two all-day face-to-face participatory workshops were carried out with users to test P-STEP prototypes. Figure 1 highlights how the discussion groups fed into the workshops. An outline of the workshop aims and topics used can be found in Additional File 3.

Research associates of the teams and project personnel attended the workshops and interacted with users on the day. The first workshop was attended by eight members of the design teams and nine users, and the second workshop by seven and five, respectively. Through a process of presentation, observing, and documenting the users’ experiences, the developers were able to assess what was working within the app, what needed to change, and what may require further investigation and user input to help. The workshop sessions used ‘stories’ created by the app team to frame the discussion.

**Fig. 2:**
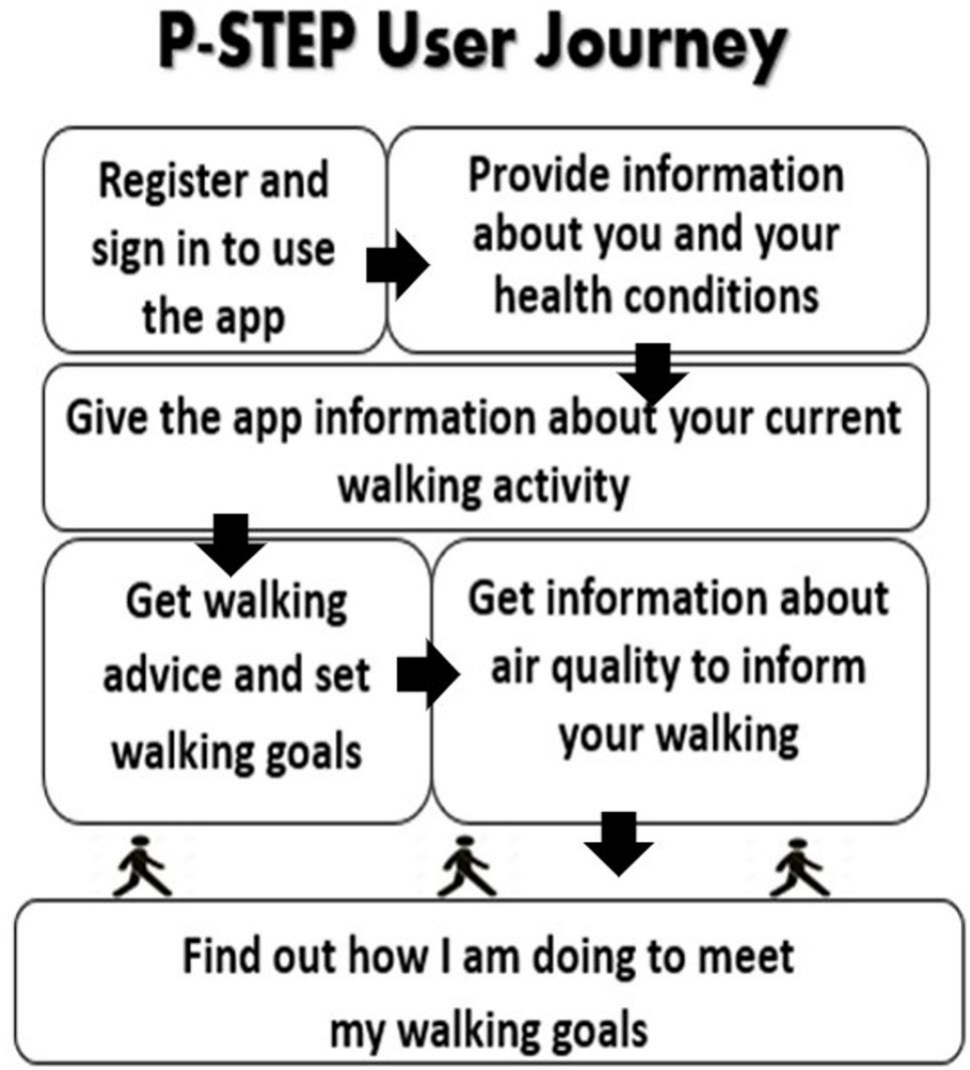
The PSTEP user journey

A user story is an informal way to present software features to users to support the development of system requirements for mobile phone apps. This enables development to be broken down into discrete tasks to support design iterations [1, 12]. These stories were incorporated within a simple P-STEP user journey (see Fig. 2), which saw individuals use different aspects of the app and provide feedback on their experiences and preferences according to their varying requirements.

### User experience of FUN

The first part of this section has described the methods adopted by FUN to involve users in the design of P-STEP and to impact the design. However, as discussed earlier, FUN also tried to create a positive experience for users, one that was empowering, positive and about human relationships. In order to capture this experience, users were asked to write about their experiences (as authors) to contribute to this article. A narrative approach allows the lived experience to be provided in a powerful way [30], and enables insight into how users made sense of their experience of being involved in P-STEP and comment on the approach undertaken by the team. Their narratives were collected after the development. Seven users participated and were also involved in reviewing and commenting on the article. We would encourage readers to read the full narratives from users (see Additional File 4).

Quotes from these narratives have been incorporated into the manuscript in a novel way. Links are made from users’ narratives to the UK Standards for Public Involvement [25] to construct a matrix of views on the quality and consistency of involvement in P-STEP (see Table 6 below). In turn, users were asked to comment on this matrix and whether it provided an honest account of their experiences. Feedback included:

*It comes out of what we said*

*We were open to say what we wanted*

*The table shows the honesty of the involvement. You have put in quotes that show the standards have been met but none of us*,* when writing*,* used these standards*,* so it has all been natural*,* and I really like the honesty of that*

## Results

### User impact on the P-STEP design

**Fig. 3:**
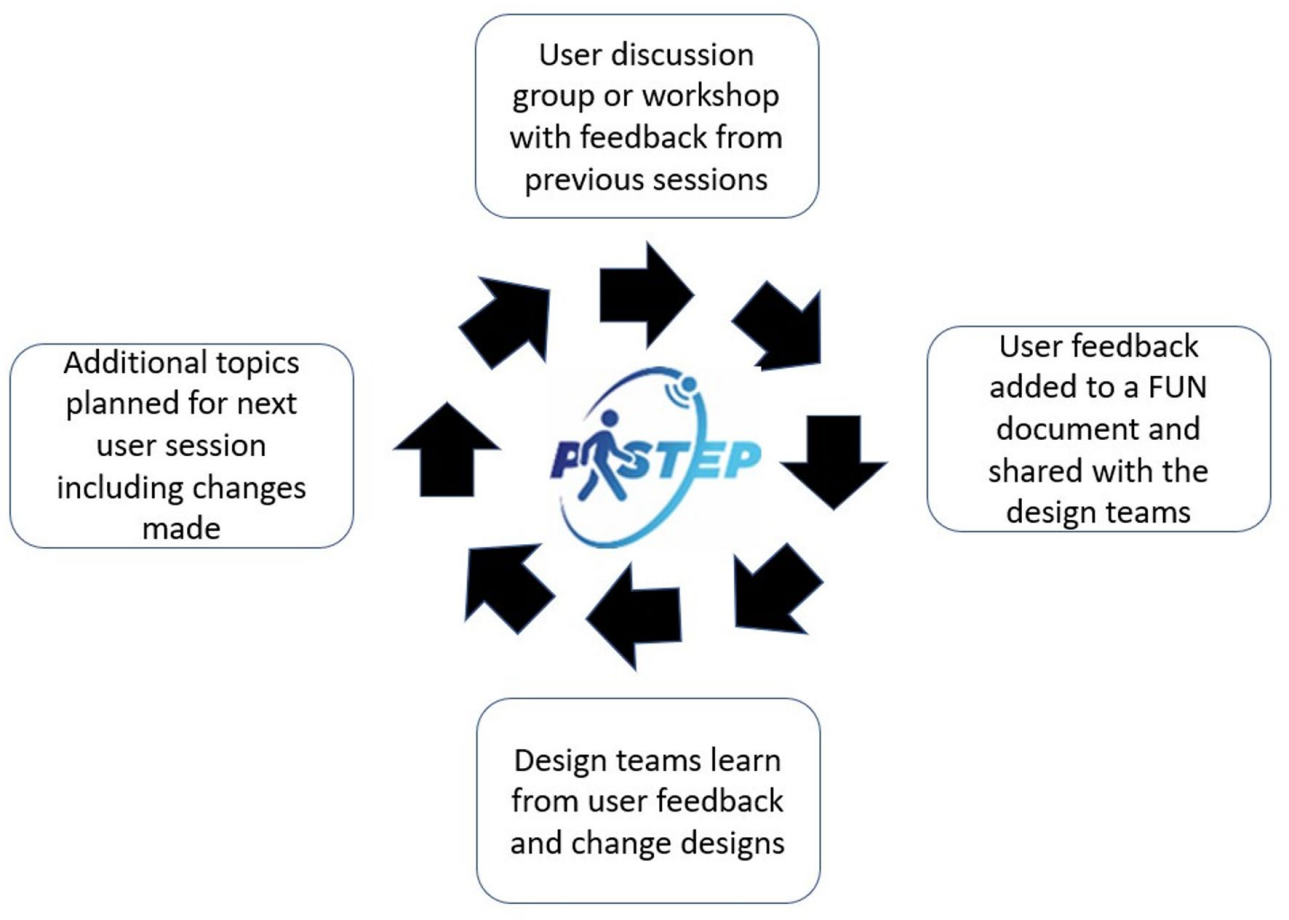
The P-STEP feedback loop

A challenge for the FUN approach was to ensure that users’ perspectives informed the development. The three weekly cycles of the FUN approach enabled the different teams to learn from each discussion. After each session, the teams were able to consider what was usable and acceptable to the users, obtaining consistent feedback via the FUN documents provided after each discussion. This created a ‘feedback loop’ between users and the teams (See Fig. 3).

Users were able to influence the design through this process. An example was the P-STEP ‘home’ screen, which users wanted to be simple and easy to use. Comments from users included:

*It doesn’t want to be too wordy*,* you know….try and keep it as short and as brisk as possible because you’re talking about looking at a phone…you don’t want lots and lots of writing where you’re having to scroll down and things like that because you know that puts people off.* [P-STEP user]

*Accessibility issues must be paramount*,* I think this in all these things*,* whether we’re talking about the choice of colour or contrast*,* size of font*,* etc.[P-STEP User]*

**Fig. 4:**
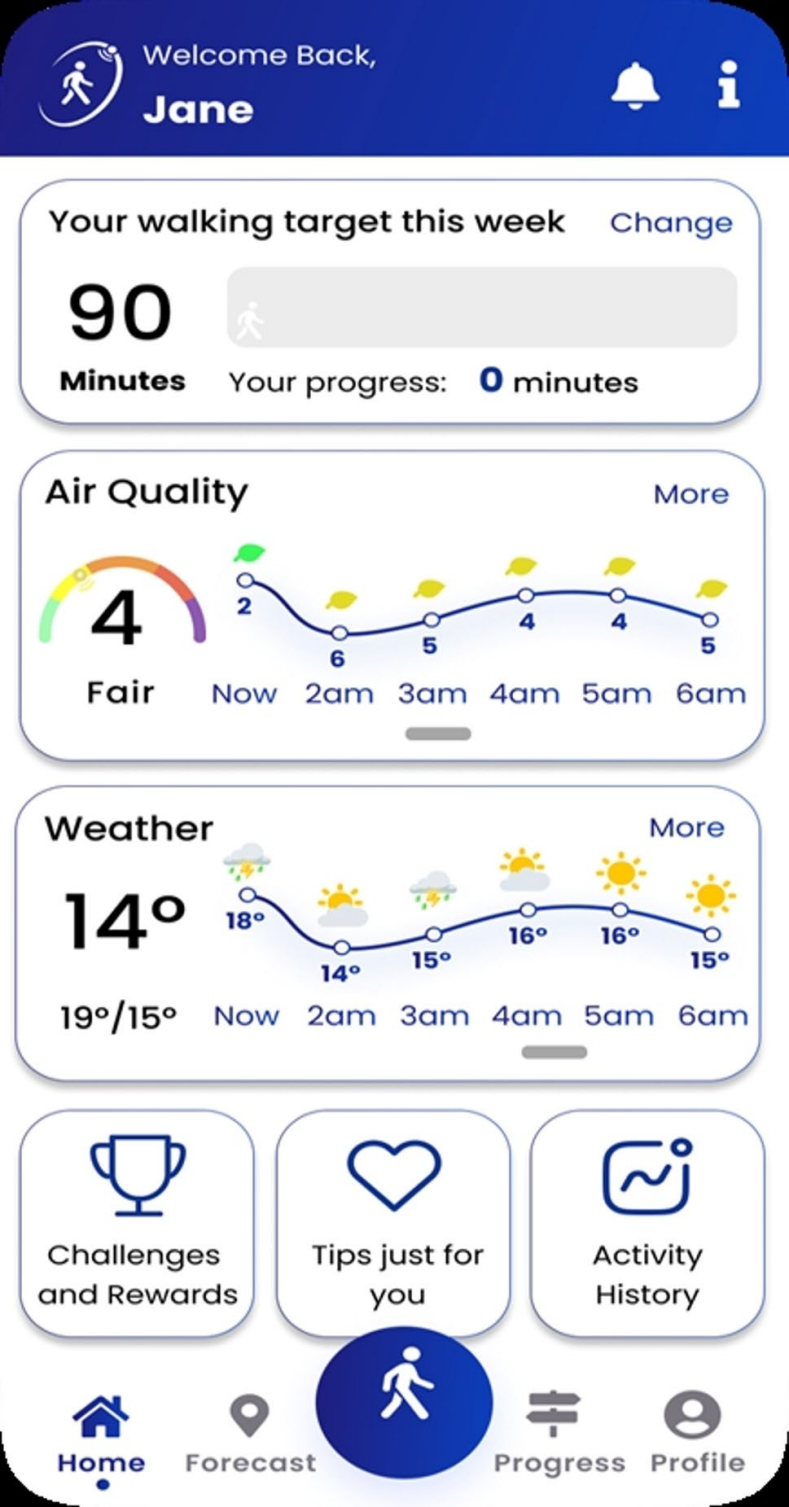
P-STEP prototype home screen

Some of the challenges of app design include reconciling the different perspectives of users and developers. Tension existed among developers wanting to create a dynamic design for the home screen rather than a basic one, and not understanding the potential drawbacks for users. Different designs of ‘home’ were shown to users and iterated by the app team until eventually mutual agreement was reached. The result was a ‘home’ screen that not only met the user requirement of accessibility and simplicity but was also viewed positively by the team (see Fig. 4). A balance was found.

Another example was the importance of messaging for those with long-term conditions. Users felt that the variable nature of their conditions could make exercise messages demotivating and patronising. Comments included:

*I can’t walk briskly*,* it would be much better for me to walk steadily for longer…I just think these messages are being sent…they’re just very generalised and they probably have been set by young…fit people and I think they’re… discriminating against those of us who are doing our best*,* but we can’t meet these …targets. [P-STEP User]*

*I would find that extremely patronising. I’m not 12. Those little stickers. I used to give those to four-year-olds…with stars and smiley faces….You know….That’s how I would feel about it. I don’t want people giving me stars or whatever. [P-STEP User]*

Messaging, therefore, needed to be tailored to help users cope with their condition and not feel disempowered by inappropriate language. This was important learning for the designers to review the style and tone of messages for people with long-term conditions. Reflecting on this article, one user said…

*I think this is a good illustration of the types of input made as things were going along and how this made a difference to the end product. It wasn’t just tokenism; it shows how things went on*,* and the app incorporated what people were saying. We gave that input*,* the team went away*,* tweaked things*,* and came back*,* and this is why we felt our inputs were valued because we could see this happen as a result of what we said. [P-STEP User]*

The participatory workshops enabled the ‘trialling’ and ‘testing’ by users of early and later prototypes, enabling the developers to learn about the strengths and weaknesses of their design, and for users to see how the teams had responded to their ideas. In this respect, users were seen as part of the ‘design’ teams helping shape the direction of their designs. All core members of the different teams attended both workshops, enabling users to meet them and to be observed interacting with the early prototype.

Users were positive about the progress made in designing the app:

*…I have been very impressed today by the app and the progress the team have made. I would be very happy to use an app like this*,* as for someone with a health condition*,* it would help me monitor my fitness and the environment in a way that other apps I use do not. The prototype is very comprehensive*,* and it would appear that it will be easy to use once initial navigation can be familiarised. Looking forward to the final version. [P-STEP User]*

Workshop 2 enabled this positivity to be tested further using recognised scores. Table 2 shows the scores from users based on a System Usability Scale (SUS) questionnaire [24] used during this workshop. The average score of 68 is considered good, with all P-STEP users producing scores above this average. Users appeared to enjoy using P-STEP and found it usable and acceptable.

**Table 2:** The usability scale

User 1	User 2	User 3	User 4	User 5	User 6	User 7
72.5	97.5	70	75	80	87.5	72.5

An additional questionnaire, the P-STEP Engagement Short Scale, was used by the app team to consider if users were ‘engaged’ by P-STEP. Table 3 presents the scores for each area.

**Table 3:** The P-STEP engagement scale scores

	User 1	User 2	User 3	User 4	User 5	User 6	User 7	Average
Focused Attention	3	3	3	3	2	1	3	3
Perceived Usability	x	1	2	2	3	2	1	2
Aesthetic Appeal	4	5	4	3	4	4	5	4
Reward	x	5	4	4	4	5	5	5

Please note that User 1 did not complete the full questionnaire. All figures are calculated to the nearest whole number

Three is average, and so any score above this would be seen as good. The responses, although generally positive, saw more feelings of ambiguity, with Perceived Usability scoring below average on this scale. Perceived usability is a user’s subjective assessment of how easy or difficult a product is to use [31]. This is seen as different from actual usability, which is often measured through standardised questionnaires such as the System Usability Scale (SUS) (as above). Using the Fun Approach, a discussion group at the end of workshop 2 helped to clarify this ambiguity and saw both users and the designers consider potential changes to the app as a result. Several key changes were suggested by users and were incorporated in the design, reflecting how the FUN approach saw impact from users on the design of the app. Changes included:

- Some Bigger font sizes
- Improved input suitable for the motor skills of older people
- More intuitive onboarding to P-STEP
- Improved tutorial and support for using P-STEP
- Information to clarify the AQ Indicator, walking minutes, and pace

### Design team perspectives

Having an impact on the design of P-STEP was important to users, and while writing this article, they wanted to include some views from the design teams on the value of their involvement. Members of the team were asked to provide a short quote to summarise what they had learnt from the user’s contribution.

For one member, their engagement with users had helped them translate scientific concepts into lay language….

*The process with the user engagement really helped with providing the users with user-friendly science recommendations. It helped me realise that when advising people about something they are not familiar with (environmental conditions)*,* you need to be careful and as clear as possible. And I can use what I learn for my future projects.* [P-STEP Team Member]

For other members, user perspectives enhanced understanding of the intended use, which influenced what features to incorporate into the app for a better user experience…

*Previous research and analyses had shaped the proposal and main focus of the P-STEP app*,* but further discussion with end-users enlarged the understanding of its intended use. User feedback in this sense helped not only with aesthetic decisions such as styles and formats but also with practical decisions about the features and services that could be found in the app [P-STEP Team Member].*

The contribution of users’ lived experiences of their conditions was highlighted by the design teams as important. As one member of the health team commented…

*I valued the contribution users made to the health profile in the app. It was also great to see users using the app. I have worked on digital health packages before. The engagement on this project was very good [P-STEP Team Member]*

Design team members valued user interactions, and the value this provided to them left them wanting more…

*We could have perhaps tested the app more with users if we had more time but I would say what users contributed was very important. [P-STEP Team Member].*

All agreed that users’ involvement in P-STEP had made an impact….

*Undoubtedly*,* user involvement and feedback were essential to the design and development of the P-STEP app as one of the main goals of the project is to provide a pleasant user experience for people with long-term conditions that want to safely exercise outdoors*[P-STEP Team Members]

### User perspectives on P-STEP involvement

The FUN approach was undertaken not only to enhance the app’s development but also to foster meaningful user involvement. Linked to the UK Standards for Public Involvement [25], Table 4 captures quotes from the users’ narratives written by them after the development phases of the app (as described in the methods section). As co-authors, these quotes highlighted key aspects of user involvement which the FUN approach effectively supported with respect to the UK Standards. On reflection, we also felt the quotes reflected aspects of what good involvement should look like on digital programmes (For further discussion, see below). These aspects are also included in the table and linked to the UK Standards:

- Create the right environment for users to be involved.
- Have good facilitation and organisation to work together.
- Provide relevant communication about the project.
- Support learning and reflexivity in the project interactions.
- Give regular feedback about how involvement was making a difference.
- Make involvement empowering and purposeful.

## Discussion

For P-STEP, the involvement of users was seen as a priority. The FUN approach aimed to make users feel involved and make a difference in the design of the app. In achieving this, it made a difference to them also.

### What does good involvement look like

Based on our discussions above and informed by what users told us, we offer some reflections on what positive involvement looks like, which may be of value to similar and other projects.

#### Created the right environment for users to be involved

Creating the right environment in which to hold conversations was important for all those wishing to participate in P-STEP. One benefit of using online discussions was the safety afforded by social distancing during the COVID-19 pandemic [32]. However, involvement online also enabled flexibility in times/dates to suit members of the group. This flexibility saw regular attendance of users in the sessions, e.g., on average, an attendance of between six and nine users per discussion group. Regular attendance helped build cohesion and a sense of belonging amongst the group, which provided familiarity and less anxiety when attending the face-to-face workshops.

**Table 4:** Quotes from users linked to the UK standards for public involvement

UK Standard - Inclusive Opportunities. Create the right environment for users to be involved.	UK Standard – Working Together. Have good facilitation and organisation to work together.	UK Standard – Communications. Provide relevant communication about the project.	UK Standard – Support and Learning. Support learning and reflexivity in the project interactions	UK Standard – Impact. Give regular feedback about how involvement was making a difference.	UK Standard - Governance. Make involvement empowering and purposeful.
*I was very pleased to be involved in the P-Step. It was lovely to meet the design team. Everyone had so much patience in teaching and answering questions. I was very much included in the team on all discussions. [User 1]* *The meeting ground rules were set and emphasised at each meeting*,* and included confidentiality*,* freedom to both express thoughts and ideas*,* and respect for other participants’ viewpoints. [User 7].* *In each session*,* different participants contributed first*,* reflecting their personal concerns and circumstances….In the process*,* we started to think about the needs of others with different concerns who might use the app in future but were not in our group*,* we wanted to represent them. [User 6]*	*My overall impression was that a lot of thought and preparation had gone into the PPI partnership sessions. I found they were always very well resourced and organised ….[User 7]* *…. the welcoming voice of the leader (facilitator) greeted us in such a warm and enthusiastic way that I think most of us were instantly put at ease. [User 6]* *I thought that the technical engagement was very informative and useful. I also felt the process motivated us to analyse and think about certain aspects in a different way*,* something I was a little out of practice doing. [User 2]*	*There was a regular ongoing dialogue taking place between the team and PPI members*,* and insights that occurred after the session ended could still be expressed. This was another factor which led me to conclude that this project differed from others that I had been involved with*,* in a more positive way*,* as in previous initiatives*,* communication from the researchers had been more limited or generally absent altogether. [User 7]* *Engagement worked fantastic and everybody contributed. Support from the engagement team was very useful. They listened to every detail to amend the design and changes requested. [User 1]*	*It was a pleasure to be involved in P-STEP. We had face-to-face meetings with the design team and requested what we would like. They were brilliant and listened….The engagement worked very well. They listened to all the users on the project. The way the design team supported us worked very well (hats off to them). [User 3]* *I sincerely believe both sides learnt from each other…P-Step has been quite different – it was truly the meeting of different worlds – the knowledge and skills of the*,* usually quite young*,* technicians meeting the personal life experiences of the*,* usually older*,* individuals living with long-term health conditions. [User 5]*	*The difference with this project was that you saw the progress and were updated every month on any changes done. The final design is what I expected. We saw the final result. [User 3].* *We watched as it slowly became a reality*,* incorporating our feedback*,* yes*,* us*,* ordinary people*,* and at every turn*,* until you have something we are all proud of and left us with a true sense of pride and achievement.[User 4]* *Each week we had a different focus*,* e.g. size and colour of font*,* icons or small illustrations to make the screen user-friendly and to make sure it was acceptable to different genders*,* ages*,* and user groups. [User 6]*	*The final design was better than I anticipated*,* and it was heartening to see some of the adaptations that we had suggested had been incorporated. Compared to many past experiences*,* we could see the concrete evidence of our work. [User 5]* *It was an exciting moment when we first saw the app screen! It made it real; it suddenly felt a responsibility. To think we’d actively contribute to decisions about content*,* layout*,* and design was interesting and also daunting. [User 6]* *We owe the team our thanks for making the experience of co-production seamless and for their patience. As projects go*,* you would be hard-pressed to find a better example of how co-production should be done.[User 4]*

Having adequate resources to manage interactions online and face-to-face supported this inclusive environment. For example, online additional assistance to monitor the ‘chat’ and highlight any ‘hands’ raised enabled all voices to be heard equally and none to be missed. Setting out values and ground rules from the start of sessions also contributed to creating a positive environment for conversations to thrive. The commitment to these values from all participants led to safe and constructive conversations and supported working together.

#### Have good facilitation and organisation to work together

Working together was supported by good facilitation of discussions and well-organised involvement activities with the other teams on the project. The different sessions always had a lead facilitator who presented the topics along with members of the design teams to answer any questions from users and contribute to the activities. Good facilitation of sessions ensured the inclusion of all views but also promoted relationship building. Being prepared to listen and offering positive and constructive comments helped to build trust, respect, and confidence between users and the teams. This facilitation saw bidirectional conversations in what might be considered a ‘partnership-focused framework’ [33]. Together, all collaborated mutually and productively. A partnership was created, and as a result, greater user impact on the design of the app.

#### Provide relevant communication about the project

With FUN, there was regular communication about when discussion groups and workshops were taking place, updates about key milestones for the project and details of design changes to the app. Good communication was seen as important so that users felt valued, respected, motivated, and confident. This is essential for building effective relationships with users. A lack of communication is not good manners, undermines relationships, and amplifies power differences.

#### Supported learning and reflexivity in the project interactions

Creating a ‘feedback loop’ helped facilitate a learning and reflexive culture. This culture saw those involved with P-STEP developing a sense of ownership of their work. Good involvement seeks a positive and meaningful experience for participants [34]. An important aspect was being prepared to change the design based on user feedback [1]. The developers fully embraced the importance of users in this regard. Some writers have suggested that we should see involvement as ‘conversations that support two-way learning’ [35]. The discussion sessions and workshops enable those who attended to learn from each other, which helped overcome any differences in perspectives of age and experience between users and the design teams. For the app team, working with users became more than just about designing an app but gaining a deeper understanding of their needs. The discussions to clarify ambiguity about the ‘perceived usability’ are one example that saw users and developers learning together and from each other; a creative process where user choices and solutions for the design of the P-STEP app were properly considered and taken forward.

#### Give regular feedback about how involvement was making a difference

Mathie et al. [36] highlight that feedback is often lacking with involvement. Users, however, highlighted the importance of getting this feedback and thus understanding how their involvement helped shape decisions about the design of the app. It is important to allow time for feedback when planning involvement activities. Jurca et al. (2014) [37] suggest that both UX and agile working are human-centred and cyclical, and this mutuality provides an important mechanism to get early feedback on technology products from and to users. This was the case within P-STEP, creating a ‘feedback loop’ as described above, and using a variety of methods, including visualisations and ‘mock-up’ screens [1], which showed users key aspects of the app for consideration. Feedback was provided by physically showing updates of these ‘mock-up’ screens or wireframes, and also arranging workshops to enable users to test the screens on the early prototypes.

#### Make involvement empowering and purposeful

Users were not passive participants just commenting on design features but collaborators and active members of the development. The idea of co-design marks this shift in thinking from passive to active participants in the design process and incorporates democratic principles [38, 39]. Involvement in design (or research) can be classified into different models of working together, ranging from consultation, collaboration, and co-production [36]. The ethos of FUN was *collaboration* rather than *co-production* because decisions to include or exclude ideas from the design were ultimately taken by the project team. However, with the FUN approach, users felt able to influence the design and so create power for them. As Fischer et al. (2020) [21] suggest, it is not always clear what impact involvement has on app builds but our users did feel they were able to influence the design. In this sense, involvement in P-STEP *‘verged’* on co-production where users’ views were treated equally alongside others in the design. This supported ‘collective knowledge making’ [23], e.g. bringing people together with activities aiming to promote self-expression and collaboration *between all involved.*

Involvement is important for mHealth interventions to ensure they are used and adopted into the daily lives of users. There are numerous different frameworks and perspectives on involving people successfully in projects, whether for research or design. A review highlighted 65 frameworks for considering the impact of involvement in health research [33]. An article by Russell et al. (2020) [40] explores PPI impact in health research and considers how these frameworks can vary depending on if projects conceptualised involvement impact as a ‘means to an end’ (impact on the research or design outcome) or an ‘end in itself’ (impact on participants – if they feel enabled and empowered during the process). However, both aspects were important to the P-STEP Project. It is unlikely there is a ‘one-size’ for all projects. P-STEP saw the creation of FUN as what others have described as a ‘locally generated codesign activity’ [33]. We are confident that the FUN approach guided user involvement and made sure that their voice had an impact during the whole development process in this respect. However, we also recognise that FUN will not necessarily guarantee the success of P-STEP in the longer term.

### The challenges of using FUN on other projects

FUN is as much an idea as a practical way to involve people in digital design projects. Involvement from people with lived experiences is a very important aspect of developing any programme (research or design) and particularly digital solutions/programmes like P-STEP. The mutual learning for both the users and the developers is vital, particularly given the distinctiveness of apps and the linking of health conditions. As Astell et al. [41] highlight, coproduction requires an environment where all voices are heard. In seeking to be participatory, inclusive, and responsive to users, ‘doing with’ them, not ‘to’ them, FUN incorporated co-production principles even if it didn’t challenge any existing power relations central to a co-production approach [42]. The aim was not that users simply felt part of the project but were aware from an early stage that they formed an integral and key part with an equal role to play. Users were empowered to have a voice and became part of something greater than themselves. They developed feelings of ownership over P-STEP as their ideas became part of the design, and they experienced feelings of belonging with regular contact with other users and the P-STEP teams. In turn, this enhanced their sense of self and identity as they built a relationship with the project and shared in the creation of the P-STEP mobile phone app.

However, there can also be challenges on digital projects like P-STEP, where researchers and developers are happy to involve people with lived experience in a tokenistic rather than meaningful way. This challenge was addressed in P-STEP by having a dedicated lead who could advocate for a more inclusive and democratic way of working with users to ensure their voices were heard. This is a lesson for other projects. Involvement practice is not just about facilitation, planning, designing sessions/workshops, writing feedback documents, good communication, and providing feedback for users on their impact, but also about advocacy. The idea that involvement is easy is misplaced. Behind the scenes, there is a lot of work undertaken. Accordingly, when planning projects involving people with lived experience, projects should not underestimate the amount of work that is required to properly involve users and employ dedicated staff to do so. For FUN to be replicated in other projects, success must include setting up the approach from the start and ensuring there are resources to support this approach. Considerable effort is also needed to ensure involvement is successful in all development phases. Box 2 includes some key messages for other projects we have taken from our discussions, reflections and observations as a project team.

**Box 2:** Key Involvement Messages for other projects using a FUN approach

• Set up an involvement approach from the start of the project.
• Ensure there are adequate resources to support the approach.
• Involve users throughout the development of a product rather than just a few focus groups or workshops.
• Undertake a high level of engagement to make users feel involved and avoid tokenism.

### Limitations

This article has tried to capture the experience of users in P-STEP, which has been overwhelmingly positive. A limitation, however, is that no quotes were used from beyond the authorship team, which may create a bias in the paper, given that three members of the core user group did not contribute to the writing.

The Pollyanna Principle [43] highlights the tendency for people to remember pleasant rather than unpleasant experiences. In this respect, critical voices from users were seldom heard. Although users were asked to critically reflect on their involvement, there may have been some anxiety about criticising the work of P-STEP when justified. For example, the project team encountered logistical challenges with the practicalities of carrying out user involvement, e.g. payments were delayed due to bureaucratic barriers within the university administration.

Arguably, those involved in P-STEP were also the so-called ‘usual suspects’ [44], and far from challenging power, the project involved those who already had substantial social capital. For example, some members of the group had professional backgrounds which would have framed their experiences. Although the project did involve a diverse set of users in terms of gender and ethnic identity, there can be significant barriers to people getting involved in health research or design projects. Barriers exist for some groups to access and use digital technologies, often those groups who may benefit the most from these health interventions, like older people, poorer and the less educated [45].

Reporting on this work has also been challenging, given the purpose of academic papers and their target audiences. User involvement in the design of digital mHealth interventions has tended to focus on the process and not the experience of users [46]. Projects often only involve users commenting on article outputs [41] rather than writing about their experience. In this paper, we have tried to capture both the process and user experiences by including users’ written narratives, ensuring that the voices of people with lived experience are present. We hope this paper helps take this aspect forward and adds to this literature, as well as showcasing what we have done.

The inclusion of user perspectives (whether written or via other methods), in our view, could be an important addition to tools that seek to report on involvement, such as GRIPP2 [47] (see Additional File 5) and highlights a difference between our approach, compared to others. Since working on P-STEP, we have also become aware that tools exist to capture involvement impact throughout the project, such as the Public Involvement in Research Impact Toolkit (PIRIT) [48]. The FUN approach, in our view, does share some similarities with PIRIT in collecting ‘in the moment’ data on involvement activity. Although P-STEP did not use these tools, FUN did follow a similar process where user involvement was reported in the ‘FUN Document’ (see Additional File 2). However, we would argue that FUN is more than an impact tool but also a ‘way of working’ as demonstrated in this article. The approach is pragmatic, open and flexible, and could be tailored to other types of projects. Evaluating the impact of involvement is a complex area, and it is difficult to balance the need for a consistent approach with the flexibility required for projects to ensure practical administration and inclusion. In this respect, FUN can offer some useful guidance and ways to capture involvement activities, impact and the experience of users.

### The future of P-STEP

A recent evaluation of the app, with a wider cohort of users, has successfully measured the usability and acceptability of the P-STEP app and found it to be satisfactory [49, 50]. Further development is continuing at the time of writing to enable P-STEP to be linked to wearable devices and to be used on Apple mobile phones (the original was Android only). Research studies are also being prepared to test whether using P-STEP will help reduce pollution exposure during walks.

## Conclusion

A great deal of time, money, resources, and effort go into designing digital interventions such as P-STEP. User involvement is vital to increase the chances that such innovations will be adopted by those who most need them. This article wanted to showcase the process that saw people with lived experiences impact the design of a mobile phone app. Involvement evaluation should highlight the experiences of users as well as the process of involvement. When developing any project (design or research) and particularly digital solutions/programmes like P-STEP, it is important to understand what good involvement should look like, the resources needed to make this happen, and the difference it could make. We hope that this article shares what we did and learnt. The inclusion of user narratives, we hope, also showcases how other projects could involve users in the reporting of their experiences.

## Supplementary Information

Below is the link to the electronic supplementary material.

Supplementary Material 1

Supplementary Material 2

Supplementary Material 3

Supplementary Material 4

Supplementary Material 5

## Data Availability

Included in additional files.

## Abbreviations

AE: Aesthetic Appeal
AQ: Air Quality
COPD: Chronic Obstructive Pulmonary Disease
DHSC: Department of Health and Social Care
FA: Focused Attention
FUN: Feedback User Needs
GRIPP: Guidance for Reporting Involvement of Patients and the Public
HCP: Health Care Professionals
LTC: Long Term Conditions
mHealth: Mobile Health
NIHR: National Institute for Health and Care Research
PIRIT: Public Involvement in Research Impact Toolkit
PPI: Public and Patient Involvement
P-STEP: Personalised Space Technology Exercise Platform
PU: Perceived Usability
RW: Reward
UES-SF: User Engagement Scale – Short Form
UK: United Kingdom
UX: User Experience
WHO: World Health Organisation

## Footnotes

1 For more information see https://business.esa.int/projects/p-step.

2 Technical development of apps is often categorised as either Waterfall or Agile methods although in practice many projects will contain elements of both and involve users. The Waterfall method gathers user requirements and tests the product once developed whereas the Agile method uses an iterative approach to ‘test’ incremental changes suggested by users. This continues until Minimum Viable Product is created. The former is therefore a linear approach which assumes users will know what they require and want and are involved when required for the design whereas the latter assumes more of a collaboration between users and the design team and requires users to be involved throughout in. In this respect, users may be seen as part of the design team which help to shape the direction of the design. At the end of each iteration users can see, comments and if possible, use what has been developed [1].
