# Supplementary material for: Putting FUN into involvement: feedback user needs in the design of a mobile phone app for people with long-term conditions
Source: Res Involv Engagem. 2026 Jan 24;12:15. doi: 10.1186/s40900-026-00837-0 (PMC12870314; doi:10.1186/s40900-026-00837-0)
Supplement: Supplementary file 1 — Supplementary Material 1 [file 40900_2026_837_MOESM1_ESM.docx]

**Additional File 1: Table Summary of Discussion Groups**

| **Session and Topic** | **User** | **Methods** | **Impact/Influence on App Design** |
| --- | --- | --- | --- |
| 1. 1. Introductions | n=10 | This was an introductory session to P-STEP for users. This started with an ‘ice breaker’ where all participants, including those from the involvement team, were asked to say when they first started using a mobile phone and computer and if they currently use apps. | This provided an opportunity for those attending to speak and the involvement team to get an idea of technology use and knowledge within the group. This ranged from those who had been using computers and mobile phones for several years to more recent adopters. There were a few laughs as each person recounted their first experiences. |
| 1. 2. Health Recommendations | n=7 | The group were asked to comment on health questions and precautions that were considered for the app:   - The group reviewed the terminology of the health recommendations, precautions, and tips. Minor alterations were made - The group suggested that links to information about conditions should be included - The group wanted symptom support and understanding in the app | The discussion helped to develop and refine these recommendations. In turn, this information was given to the design teams to develop mobile phone wireframes to capture users’ health information and highlight any precautions required for their conditions when walking. |
| 1. 3. Planning Walking Routes and Getting Information About Air Quality, Temperature, Heat and Pollen Levels | n=9 | The group were shown several different options and asked to comment on what they liked or didn’t like about these. Options included:   - Getting a forecast and not planning a route but just walking. - Setting a route with a start and end like a satnav. - Saving routes as favourites, which they could access later. - Being offered recommended routes like ‘TripAdvisor’ | There was a rich discussion and different views expressed by those who attended. This gave the design teams a lot to think about. The comments helped the team decide on the best way to include walking routes in the app. The group wanted the flexibility to see general location pollution and specific route pollution:   - The group wanted information about specific routes, e.g., bus stops and the ability to add comments about a route - The group liked the idea of saving routes and having recommended routes |
| 1. 4. Motivation | n=6 | The design teams wanted to understand more about what motivated users to go walking and how the app could support this. The first part of this session was a general discussion about internal and external motivation, followed by examples of motivation screens from other mobile phone apps for users to comment on. The examples discussed for motivation included:   - Setting goals - ‘Tracking’ progress - Getting rewards - Using games, music and education - Receiving positive messages - Sharing their progress | Again, there were a lot of rich discussions and different views expressed by those who attended, including being motivated by being able to track their progress, having some positive messages and sharing their progress with others. This included that:   - Messaging should be age-appropriate and sensitive - especially in times of illness - An option to turn off messages should be included - Could goals be set using creative ideas, like how many ‘Ben Nevis’ you have climbed - Users suggested including a progress page that could be shared with family, friends and any health providers if they wished   The comments helped the design team to consider how to include motivational aspects within the app. |
| 1. 5. Visualisations of P-STEP | n=10 | In this session, the design team had been busy designing screens for P-STEP, considering previous user feedback and wanted to present different options to users, e.g., whether P-STEP should use photo images or illustrations. This included quickfire questions about the design, colours, style, and images being considered for the P-STEP app. Those who attended were presented with different options and asked to vote. | This session enabled the design team to agree on what images to include in the early prototype of the P-STEP app.   - The group did not want stereotypical athletic people pictured in the app - Slightly more users were in favour of images over illustrations to include in the app. |
| ***This was followed by the 1^st^ workshop to test an early prototype.*** | | | |
| 1. 6. Messages and Notifications in P-STEP | n=9 | Messages and notifications had not been included in the early prototype, and to start a discussion about these, a case study of John, who had asthma, was presented to users with examples of the messages he could receive from P-STEP. Messages were:   - Informative (for example, how to use the app) - Reminders (for example, days to go walking/Air Quality/Weather) - Progress-related (for example, how well a user was doing) - Motivational (for example, encouraging users not doing well to do better) | This session provoked some lively discussions about the tone and style of the messages that were presented. The group highlighted both positive and negative aspects of the messages and recommended changes for the design teams to consider:   - A ‘gentler’ rather than more direct messaging and notification for users. - Keeping messages friendly and upbeat - Having concise rather than wordy messages and notifications - Ensuring messages and notifications reflect the diversity of different conditions and do not demotivate users |
| 1. 7. Setting Goals and Home Screen Ideas | n=9 | This session saw a change from general topics to more specific issues for the design teams to inform the finished prototype. For example, the group were asked if they preferred to set their own goals or have P-STEP do this. | The discussion highlighted different responses, but a consensus emerged that users needed both a baseline set by P-STEP and the ability to change this. The group also wanted to remind the designers of the importance of accessibility, like contracted colours and larger text sizes in any design for the home screen. |
| 1. 8. Reminders, Messages and Visualisations | n=10 | This session returned to the previous topics discussed. Previous discussions had highlighted the importance of any messaging, and the design teams wanted views on several health and environmental messages, along with opinions on additional visualisations within the app prototype. | Users again emphasised the importance of the tone and style of the messages (including avoiding technical language) and having the option to customise when they would be received. An important development was to suggest that messages need to be linked to specific conditions. Users were also highlighted that the health and environmental messages appeared contradictory, meaning the design teams had to further consider how best to communicate advice to users who will be using the app. |
| 1. 9. Home Screen, Routes and the P-STEP Website | n=9 | Like the previous two sessions, specific topics were presented to the group for their comments to finalise the prototype (Version 1) before a 2^nd^ Workshop, when users would try and test this version.  This included further discussion about the ‘home’ screen and terminology for route information.  In addition, users were asked to comment on some early designs of the P-STEP website and what information they felt should be included on the website. | Users were able to emphasise that the ‘home’ screen should be simple and accessible for users, and any route terminology should be meaningful and provided in lay terms. They also suggested that further instructions be included to support users with P-STEP. Users also had various suggestions for the website:   - Include general information about pollen, mould, humidity, and air quality - Break the information into short chunks using bullet points rather than large blocks of text - Have an initial page that tells users and clinicians what P-STEP is, promoting the walking and air quality aspects - Consider separate pages for users and clinicians to overcome technical language |
| 1. 10. Time for Reflection | n=9 | The session was a reflective session held close to Christmas and allowed the group to celebrate the work done over the year. Some photographs of the 1^st^ workshop were shared, and all enjoyed seeing and discussing these. The group were shown a video of the new prototype (Version 1), which highlighted the progress made by the design team and the plans for the 2^nd^ workshop were discussed. There was also a discussion of the future of the P-STEP project and the end of the group’s contribution and involvement in the project. | It was suggested that the group may wish to contribute to this article, and volunteers were asked to let the team know if they wanted to do this. Seven users did so. |
| ***This was followed by the 2^nd^ workshop to test the revised prototype.*** | | | |
