# Supplementary material for: Putting FUN into involvement: feedback user needs in the design of a mobile phone app for people with long-term conditions
Source: Res Involv Engagem. 2026 Jan 24;12:15. doi: 10.1186/s40900-026-00837-0 (PMC12870314; doi:10.1186/s40900-026-00837-0)
Supplement: Supplementary file 2 — Supplementary Material 2 [file 40900_2026_837_MOESM2_ESM.docx]

**Additional File 2: Example of FUN Document**

**P-STEP User Engagement Core Group Session 8^th^ November 2022: 10.00 am-11.00 am**

1. **Attendance**

The session was conducted online using Microsoft Teams:

| P-STEP Team |  |
| --- | --- |
| Users attending | 10 Users attended. |

1. **Background and discussion topics**

The main purpose of this session was to consider the messages provided within the P-STEP app. Other topics also included reminders and visualisation updates of the Home screen.

1. **Findings and feedback**

| **Question discussed:** | | | | |
| --- | --- | --- | --- | --- |
| Users were asked if they preferred explicit or gentle walking reminders in P-STEP and if they wanted to set times for reminders. | 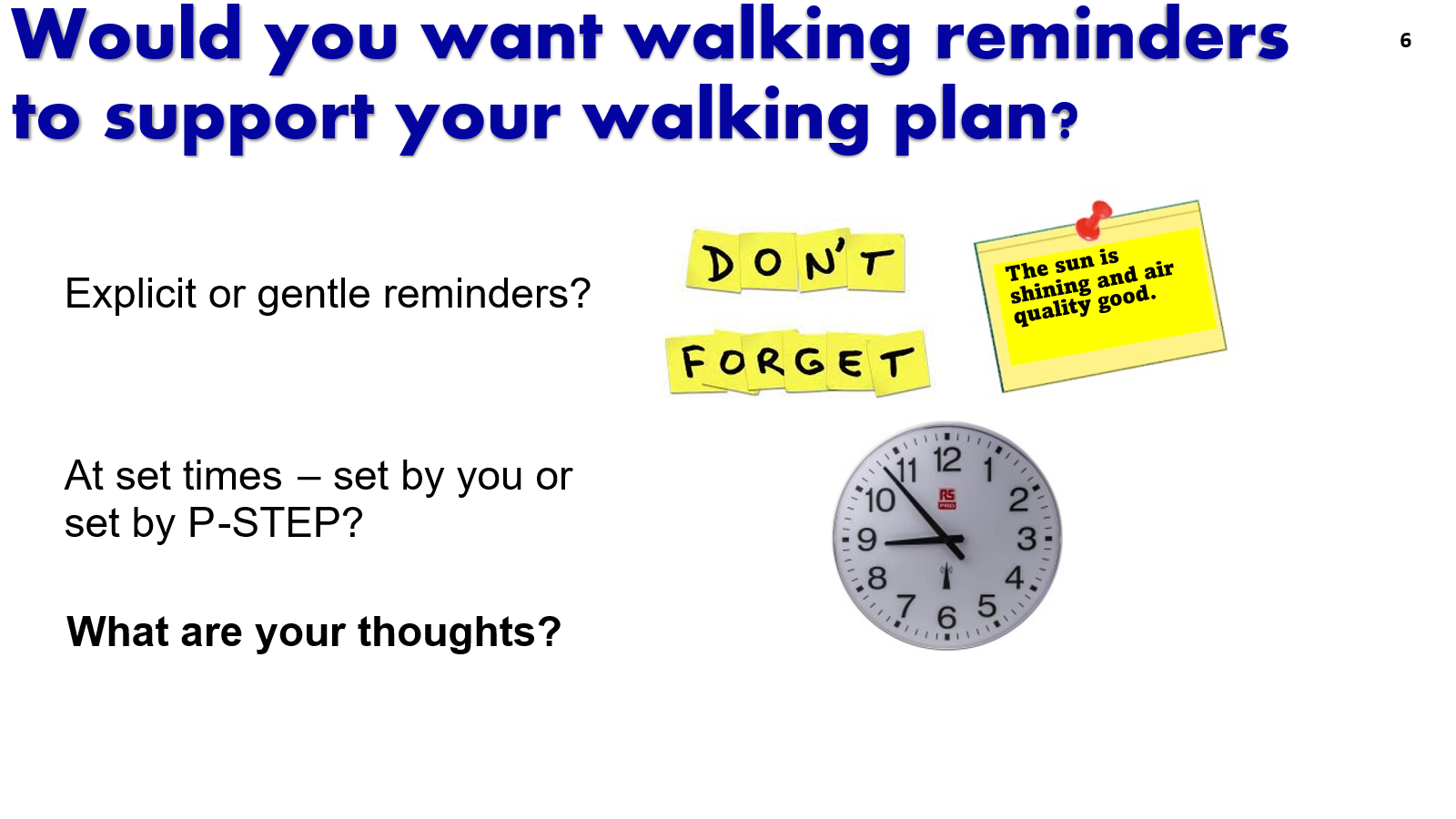 | | | |
| **UE discussion** | **Areas for consideration/development** | **Who** | | |
| Users welcomed the idea of having walking reminders. Several users suggested a gentle reminder linked to the weather conditions would be useful. Suggestions included taking an umbrella if the forecast was rain or advising to wear waterproofs.  All users felt the weather would be an important aspect to include in the walking reminders.  A user raised the issue of sending continual reminders if a user became deceased. One suggestion was ensuring users interact with or ‘accept’ some of the reminder messages.  Several users wanted to customise the times of the reminders to suit their situations – for example, if they were on holiday and wanted to change the time they received one. | Users want gentle reminder messages linked to weather forecasts.  A check is needed to stop reminders if P-STEP is not being used.  Provide an option for users to customise the times when they receive reminders. | AQ and App Team. | | |
| **Question discussed:** | | | | |
| Users were shown three different health messages and asked to give a ‘thumbs up or down’ to these. Those with thumbs down were asked for their reasons why. | 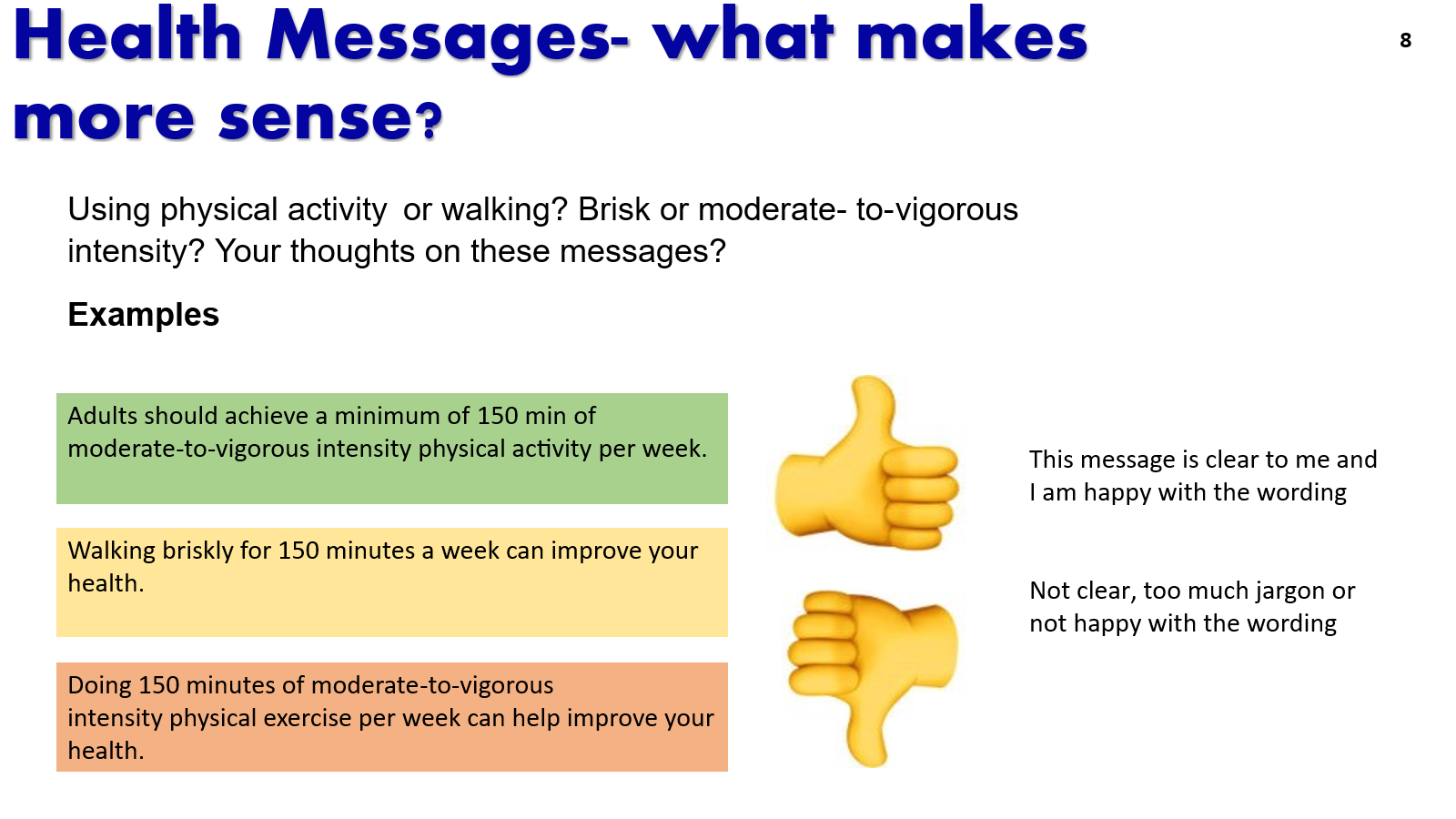 | | | |
| **UE discussion and voting**  **M=Message** | **Areas for consideration/development** | | **Who** | |
| M1 - 6 users ‘thumbs down’. 4 users ‘thumbs up’.  M2 - 3 users ‘thumbs down’. 7 users ‘thumbs up’.  M3- Discussion only  Users discussed the difficulty of defining ‘brisk walking’ and ‘intensity’, and their ability to do 150 mins of walking.  One user felt that the 150 minutes was a useful goal but they may not always be able to achieve it.  The discussion highlighted individual differences in the way their conditions impacted them, and users felt P-STEP needed to accommodate these differences in the messaging. | Enable messages to be tailored to individual conditions, considering ‘brisk’ or ‘intense’ as subjective terms.  M1 - Terms like ‘should’ need to be avoided.  Users preferred message 2 of the three highlighted. | | Health Team | |
| **Question discussed:** | | | | |
| Users were shown messages that used medical and technical terms, e.g. for diabetes and were asked to give a ‘thumbs up or down’. Those with thumbs down were asked for their reasons why. | 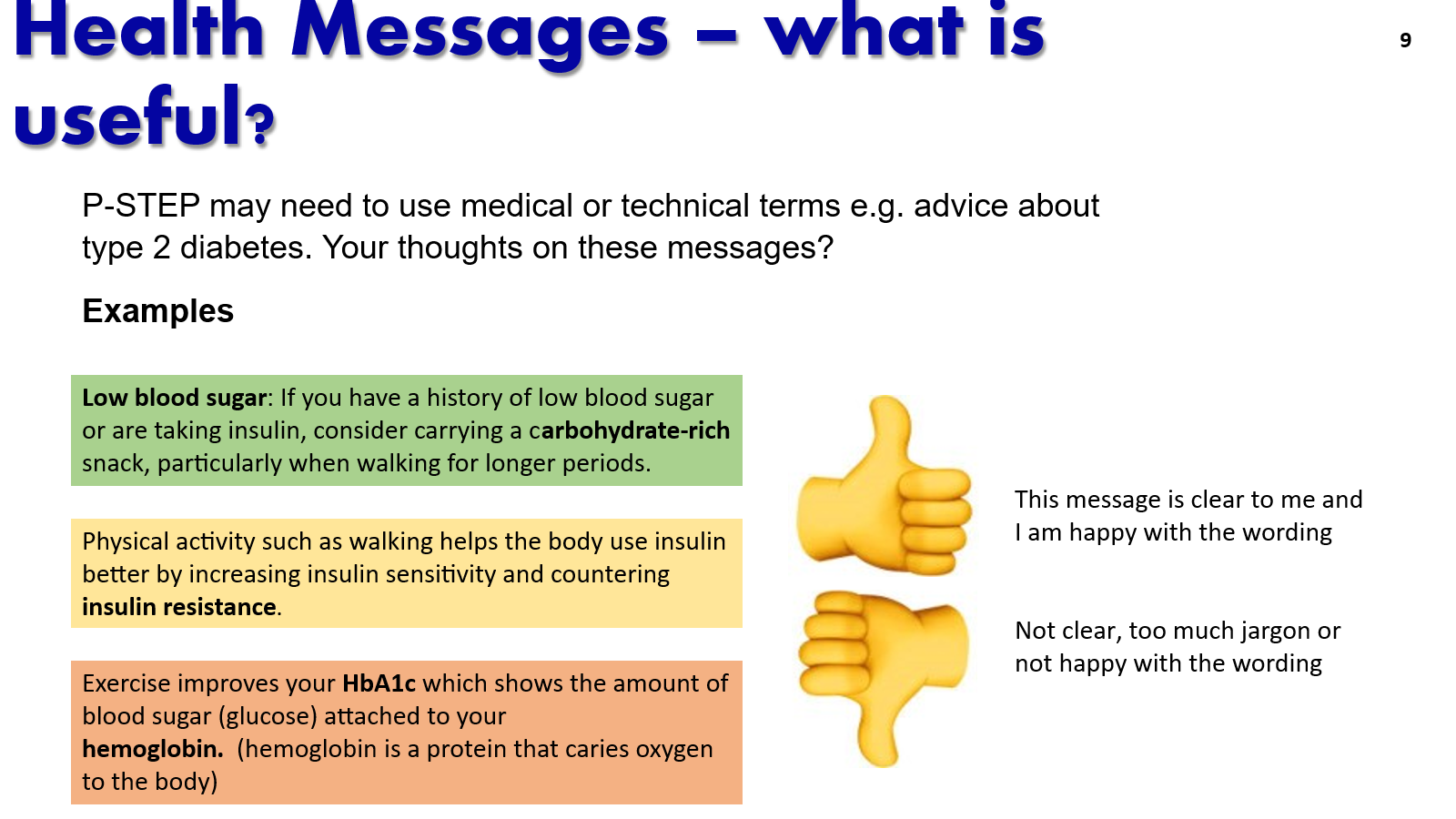 | | | |
| **UE discussion**  **M=Message** | **Areas for consideration/development** | | **Who** | |
| M1 - All thumbs up  M2 - 1 user ‘thumb down’. 9 user ‘thumbs up’.  M3 - Discussion only  M1 - One user suggested including an example of a carbohydrate-rich snack would help to clarify what this means.  M2 and M3 - Several users felt that messages should avoid medical/technical language.  [Design team members] highlighted that these messages are for educational purposes and would appear in the user’s profile. | M1 - Consider examples of carbohydrate-rich snacks  M2 and M3 - Review messages using technical/medical language and try to avoid or explain these terms. | | Health Team. | |
| **Question discussed:** | | | | |
| Users were shown messages that offered advice about Nordic walking, smoking and elevations and asked to give a ‘thumbs up or down’. Those with thumbs down were asked for their reasons why. | 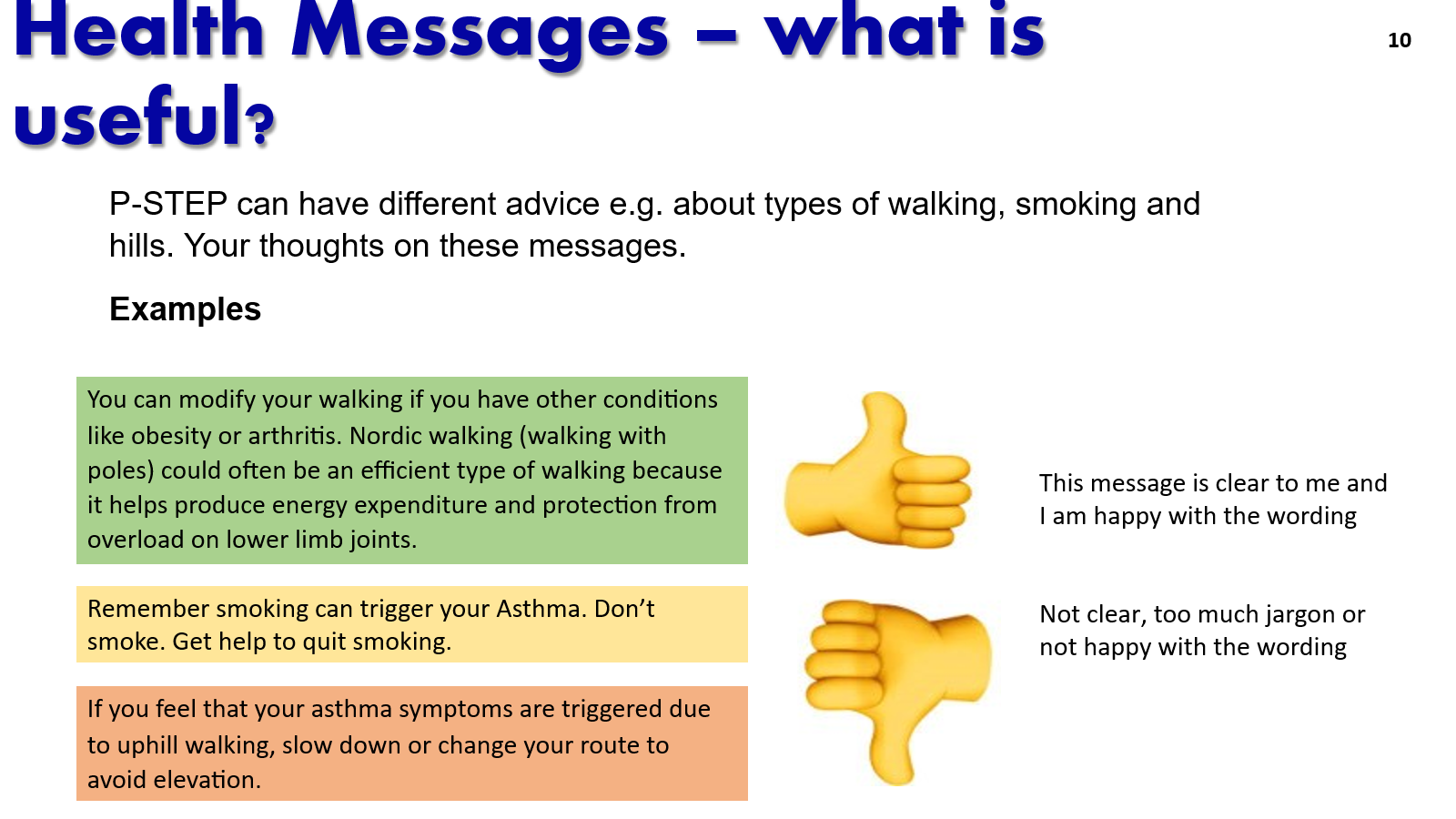 | | | |
| **UE discussion**  **M=Message** | **Areas for consideration/development** | | **Who** | |
| M1 – 2 user ‘thumbs down’. 8 user ‘thumbs up’.  M2 – 2 user ‘thumbs down’. 8 user ‘thumbs up’.  M3 - 9 user ‘thumbs up’.  M1 – One user felt the message was useful but more information was needed. Another felt the message was not useful as they used crutches, and they highlighted the strain that use of crutches can put on certain joints.  M2 - One user didn’t want to hear about smoking when using the app, and another felt that this advice would already have been provided by HCPs and may be ignored.  M3 - Users liked how the message was empathetic and acknowledged issues. | M1 – Signpost to further information about Nordic walking.  M2 - Consider if more subtle messaging may be better for those who smoke to help them give up, e.g. ‘reminding not banning’.  M3 - Messages appear to work best when empathic and not judgmental. | | Health Team | |
| **Question discussed:** | | | | |
| [Design team members] discussed with users the AQ Index and the recommendations that accompany this. Users were shown the AQ index and asked for comments about the phrasing.  Further discussion was undertaken about messages relating to pollen, and if a reminder to take medication, e.g. an asthma inhaler was useful. | 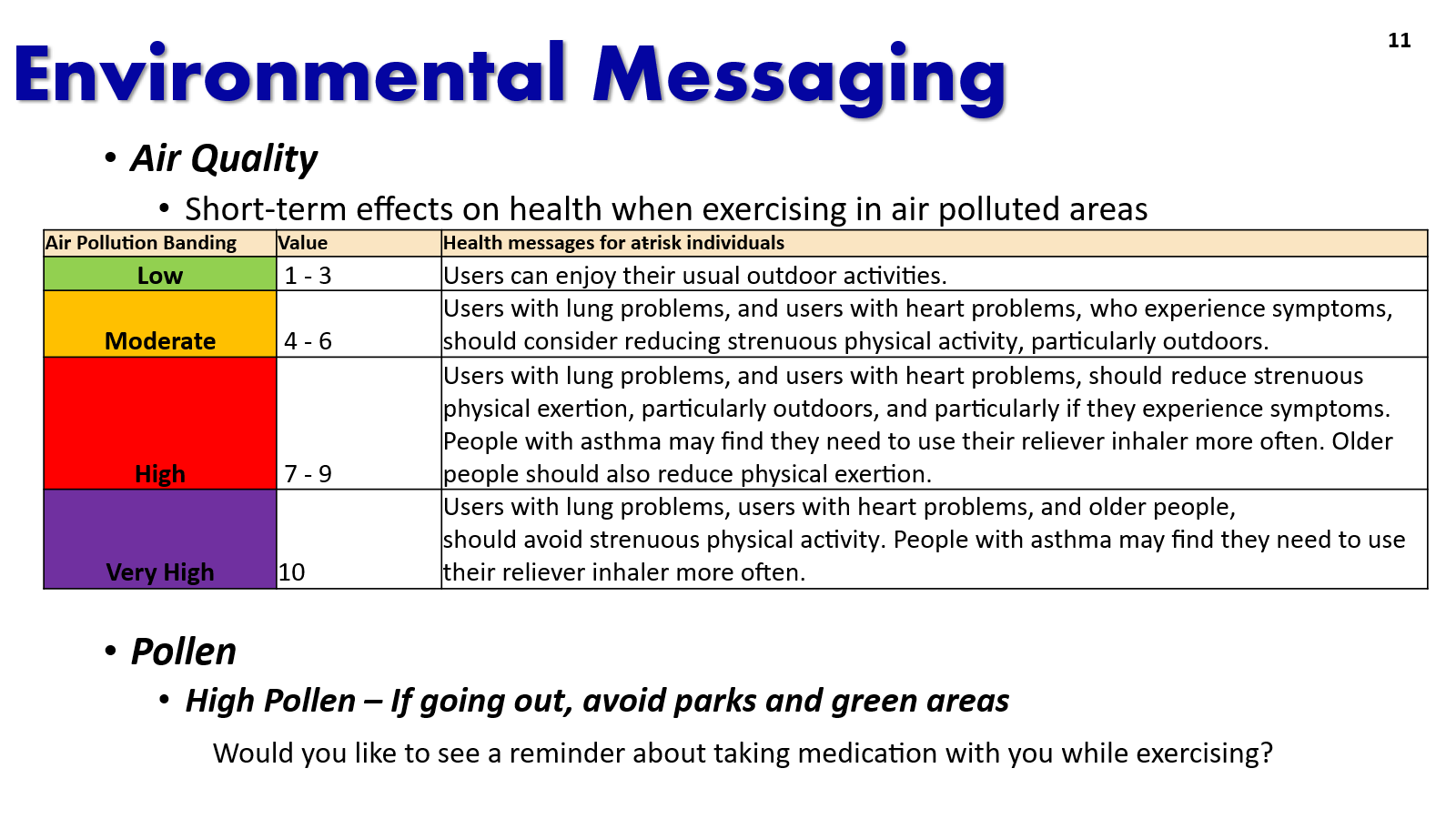 | | | |
| **UE discussion** | **Areas for consideration/development** | | **Who** | |
| One user suggested that AQ recommendations should emphasise that the advice was for ‘today’ and at that moment.  [Design team members] highlighted that P-STEP can include hourly updates if required.  Another user highlighted that the messages appeared to contradict other health advice, e.g. to walk intensively and the messages needed to work together. It was noted that this recommendation may only appear in exceptional situations, as adverse weather events (such as high temperatures) are not a common occurrence in the UK.  Users welcomed the idea of a reminder to take an inhaler but prefer a ‘gentle’ nudge. | Be clear about what AQ warning messages refer to when they apply.  Check any potential contradictions between Health and Environmental messages.  Provide ‘gentle’ reminders that prompt users to take their inhalers. | | Health and AQ Teams | |
| **Question discussed:** | | | | |
| Users were shown a Heat Index and asked for comments about the phrasing.  Users were also informed of how the current Indexes would be implemented in the app. | 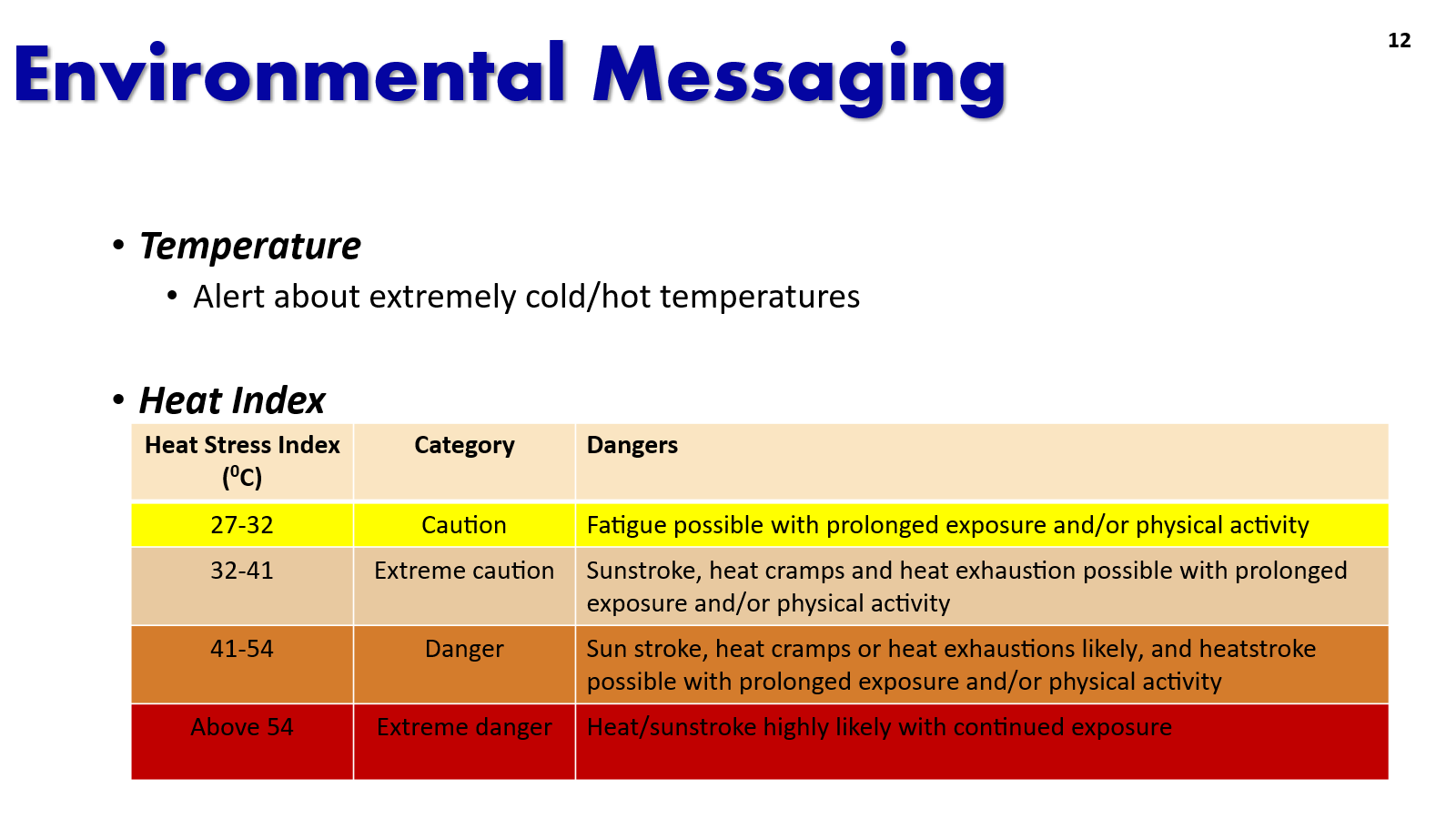 | | | |
| **UE discussion** | **Areas for consideration/development** | | **Who** | |
| One user highlighted that they could tolerate the heat but not cold and wet weather. Another advised that cold, breezy weather was more likely to impact their condition.  [Design team members] highlighted that the Heat Index also took account of humidity, and a Cold Index was not available at present to include in the app. | Users highlighted the need for a ‘Cold Index’. | | AQ Team. | |
| **Question discussed:** | | | | |
| [Design team members] showed users visualisations of the new Home screen and asked for comments about the content and what images they prefer. | 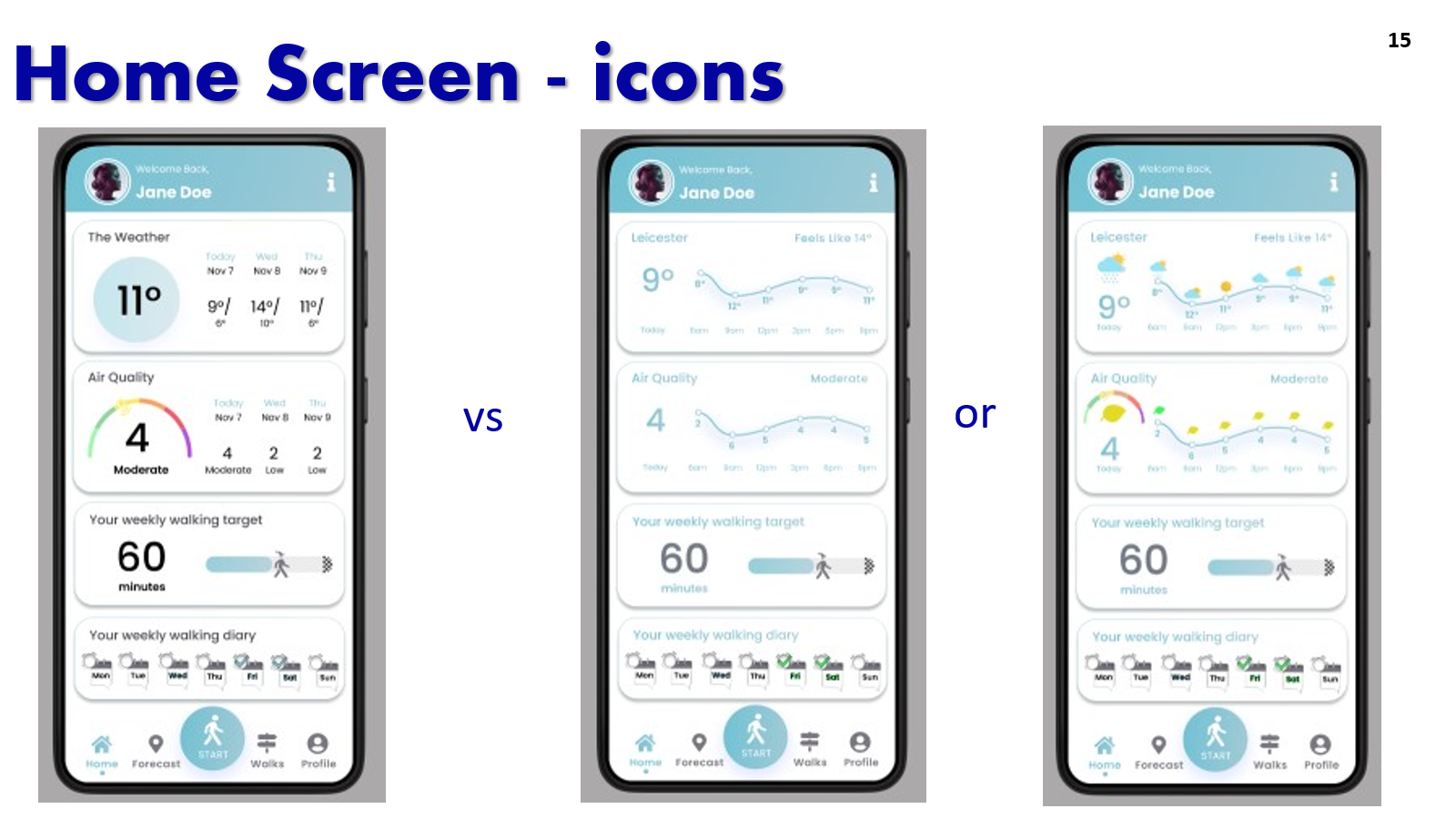 | | | |
| **UE discussion and voting** | **Areas for consideration/development** | | **Who** | |
| Users felt the content of the Home Screen was appropriate. One user asked if progress toward the walking target could be displayed, and this was confirmed.  All users voted for the visualisation on the right (third) as the preferred version. One user felt this was more accessible with her visual impairment. | Implement the ‘Home’ screen using the visualisations on the right (third).  Progress toward the walking target is to be displayed on the bar. | | App Team. | |
| **Question discussed:** | | | | |
| [Design team members] asked if users would prefer to navigate several screens to save walks or one screen with a slide-up panel. | 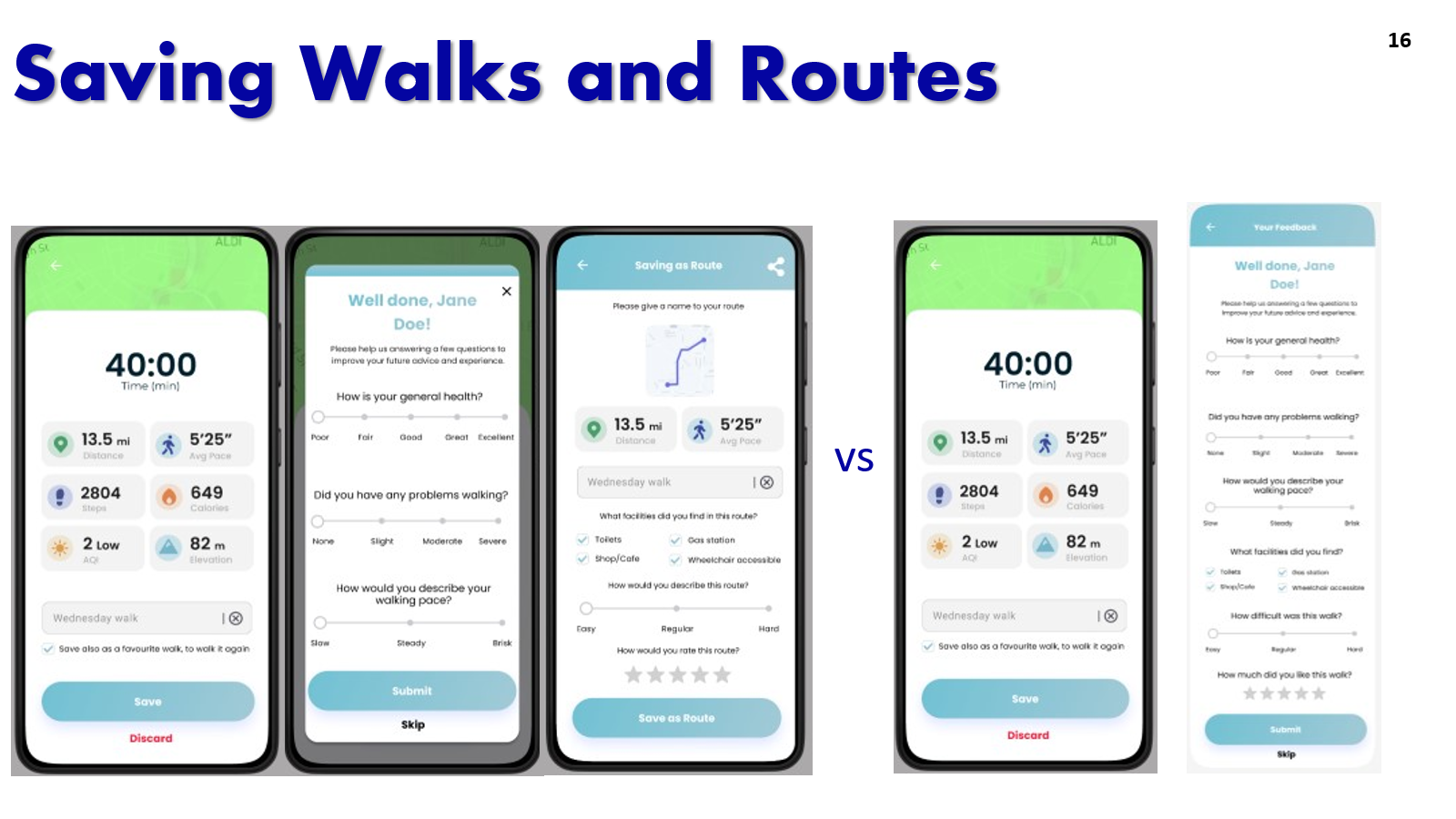 | | | |
| **UE discussion and voting** | **Areas for consideration/development** | | | **Who** |
| The majority of users voted for one screen with a scrolling function, although one user highlighted that they found scrolling difficult and would prefer the different screens. | Implement fewer screens to save/record walks but check accessibility for those who may find scrolling difficult. | | | App Team |

Other discussions

Users were informed that the next meeting would focus on the P-STEP website.
