# Supplementary material for: Putting FUN into involvement: feedback user needs in the design of a mobile phone app for people with long-term conditions
Source: Res Involv Engagem. 2026 Jan 24;12:15. doi: 10.1186/s40900-026-00837-0 (PMC12870314; doi:10.1186/s40900-026-00837-0)

**Additional File 3: Details of workshop practicalities**

1. Outline of workshop

| **Workshop 1** | **User** |
| --- | --- |
| **Testing the Early Prototype** | **n=9** |
| This workshop aimed to test the early prototype and discuss what aspects users liked and were working and/or not working. | |
| The workshop was organised around three different scenarios based on the P-STEP user journey, and one quick-fire ‘What do you prefer’ session, which involved showing users alternative visualisations to the early prototype for them to vote on.     - **Scenario One:** Inputting health and baseline conditions. - **Scenario Two:** Setting walking goals and checking air quality info. - **Scenario Three:** Starting a walking activity, saving a route and checking the profile. | |
| **Workshop 2** | **User** |
| **Trialling and Testing the Prototype (Version 1)** | **n=7** |
| The aim of this workshop was for users to have an opportunity to try and test the P-STEP app (Version 1) and highlight any further immediate developments needed before the app was formally rolled out and evaluated by a wider cohort of users. | |
| The workshop was divided into two sessions:   1. Trialling and testing the app. 2. A discussion about how well the design team had done with meeting the user journey.   Users were provided with minimal instructions and given a free hand to use P-STEP as they wished. Users were provided with a mobile phone and asked to:   - Log in to P-STEP - Set health Profile - Set the first goal. - Check forecast - Start and save a walk - Check progress/awards - Set additional goals - Check accessibility/preferences   Users could leave the workshop and walk a short distance if they wished. | |

1. Set up of tables

The ‘User’ team acted as lead facilitators for the events, starting the scenarios and prompting discussions and timings thereafter. Members of the design teams were given either a facilitator or observer role. The room was organised with three tables. Participants will be seated around the table as follows (also see photos below)

| Table One |  |  |  |  |
| --- | --- | --- | --- | --- |
| F | O | U | U |  |
|  | U | U | O |  |
|  |  |  |  |  |
|  |  |  |  |  |
| Table Two |  |  |  |  |
| F | O | U | U |  |
|  | U | U | O |  |
|  |  |  |  |  |
| Table Three |  |  |  |  |
| F | U | U | U |  |
|  |  | O |  |  |

| Code: F= Facilitator O=Observer U=User   \| 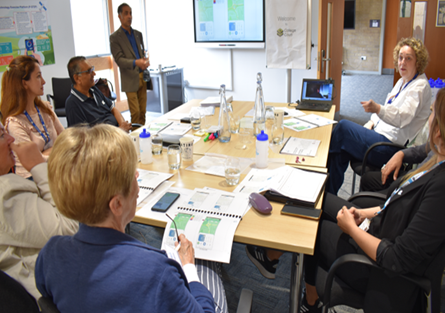 \| **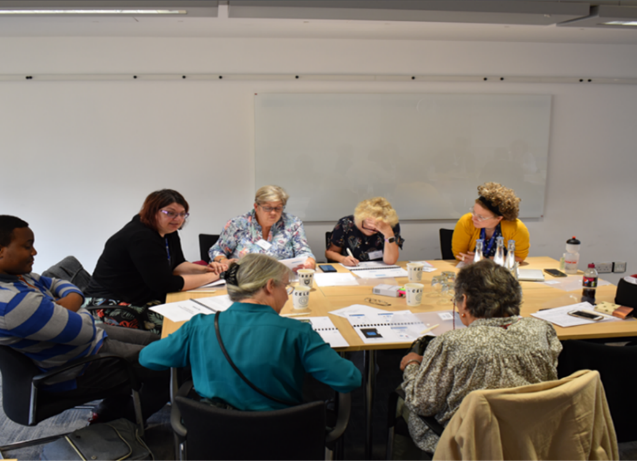** \| \| --- \| --- \|  1. Documenting users feedback   Users were provided with a booklet in workshop 1, which included images of all the current wireframes for the scenarios to document their views and a questionnaire for workshop 2 to register their views (see examples below).  Table facilitators introduced the scenario tasks to users, prompted discussions, and supported users to complete their booklets and questionnaires. Observers supported users with the tasks and documented any negative aspects of users’ interactions they felt were useful to change in the design of the app on an Observation Sheet (see below). As the table facilitators and observers were from the design teams, this enabled direct interaction with users to gain a greater understanding of what was working/not working within the app. |
| --- | --- | --- |

**Example from the user booklet (Workshop 1):**


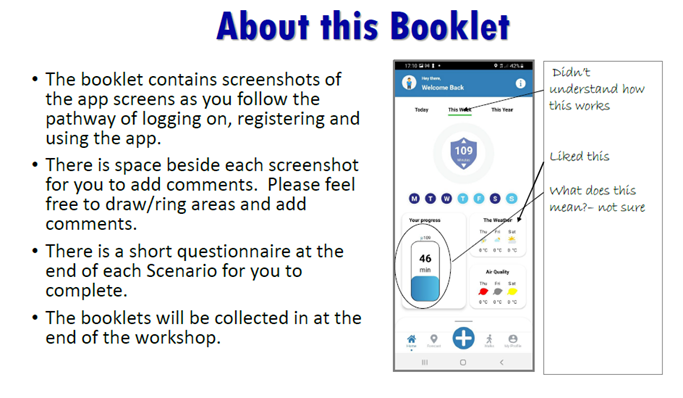


**Questionnaires (Workshop 2)**


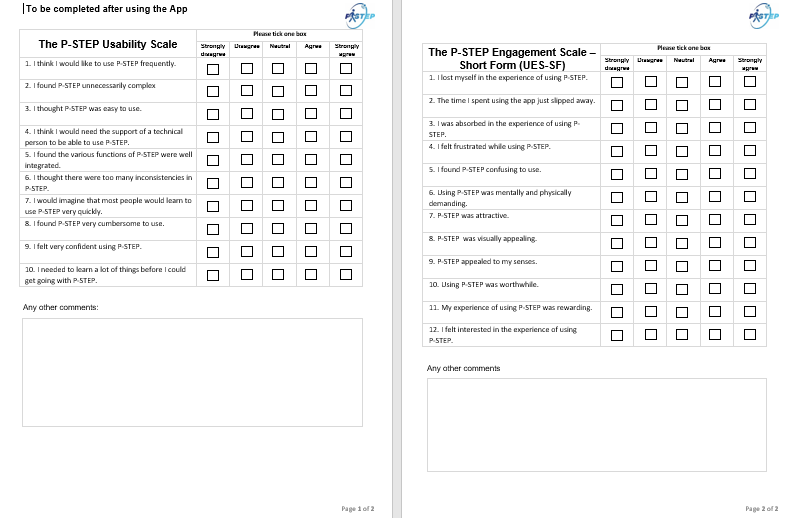


**Observers Sheet (Workshop 1 & 2)**


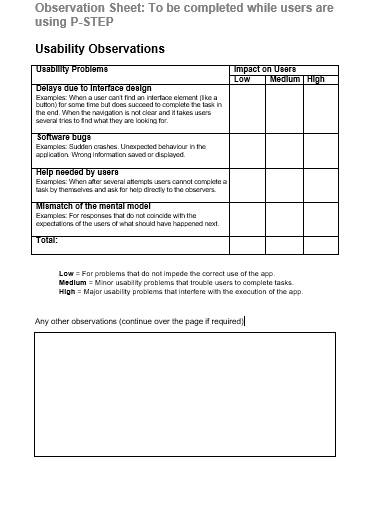

Supplement: Supplementary file 3 — Supplementary Material 3 [file 40900_2026_837_MOESM3_ESM.docx]
