# Supplementary material for: Putting FUN into involvement: feedback user needs in the design of a mobile phone app for people with long-term conditions
Source: Res Involv Engagem. 2026 Jan 24;12:15. doi: 10.1186/s40900-026-00837-0 (PMC12870314; doi:10.1186/s40900-026-00837-0)
Supplement: Supplementary file 4 — Supplementary Material 4 [file 40900_2026_837_MOESM4_ESM.docx]

**Additional File 4: User narratives**

This file provides full copies of the narratives written by members of the P-STEP user group. Users asked for some guidance to frame their contribution, and the following was provided (although they could write what they wished):

- If you felt included or not?
- Did the engagement work or didn’t work?
- Was the way P-STEP supported tech engagement useful or not?
- Were there differences from other involvement experiences with P-STEP?
- Was the final design of the app what they expected or not?
- Did they feel their contribution made a difference?

A minimum of about one side of A4 or 500 words was suggested to write but no word limit. As such, here are contributions of different lengths but all were welcomed and have been of value to writing this article.

**User 1: Contribution**

*I was very pleased to be involved in the P-STEP. It was lovely to meet design team. Everyone had so much patient in teaching and answering questions. I was very much included in the team on all discussions. Engagement worked fantastic and everybody contributed. Support from the engagement team was very useful. They listened to every detail to amend the design and changes requested. A difference from other involvement experience was that we had regular zoom meetings and were updated with each progress. The final meeting was face-to-face and we tried out the gadget which was very easy to use and successful. Looking forward for the prototype to be launched and working.*

**User 2: Contribution**

*I suffer from severe asthma so was pleased to have the opportunity to be involved in the P-STEP. I felt P-STEP would be ideal for me to encourage me to do more walking with a much more healthy outlook with regard to localised air quality in view of my condition. As this was the first time I’d used “Teams” technology, I did wonder how included or engaged I would feel. Happily, with a very good chair/facilitator, I felt both. Participants were listened to and I also felt you could get to know the other participants. When we all finally did meet up, we were relaxed in each other’s company. I thought that the technical engagement was very informative and useful. I also felt the process motivated us to analyse and think about certain aspects in a different way, something I was a little out of practice doing. Despite the varying ages of the group, I felt that the older members, me included, who are not necessarily very au fait with technical issues, were not made to feel inadequate and were always encouraged.  I have personally never used a walking app but my husband does. I did feel that this project was something personal to me rather than having to spend a lot of time “exploring” other apps out there to see if I could find one that suited me. Not all will have air quality information. I have not been involved in any projects or groups since I retired from work, and I found  it to be an enjoyable, useful and productive experience.*

**User 3: Contribution**

*It was a pleasure to be involved in P-STEP. We had face-to-face meetings with the design team and requested what we would like. They were brilliant and listened. Once a month, we had Zoom meetings to keep us up to date on the progress and how it looked. If we, the user, weren’t happy, we had a vote and suggested the changes we would like. The design team went away and amended it as requested. The engagement worked very well. They listened to all the users on the project. The way the design team supported us worked very well (hats off to them). The difference with this project was you saw the progress and were updated every month on any changes done. The final design is what I expected. We saw the final result. The user contribution made a difference in the development of P-STEP, listening to the way we wanted the layout, colours and font size. Once the prototype was activated there was always room for changes.*

**User 4: Contribution**

*I first became aware of an idea for a project into pollution some years ago. To my surprise, I was invited to help participate and co-produce the use of pollution data to map a safe less polluted walk. To help respiratory sufferers exercise free from bad pollution. All this and more in a mobile phone app called P-STEP. Working with the research team could not have been a more enjoyable experience as we all watched as our ideas were taken on board. We watched as it slowly became a reality, incorporating our feedback, yes us, ordinary people and at every turn, until you have something we are all proud of and left us with a true sense pride and achievement. We owe the team our thanks for making the experience of co-production seamless and their patience. As projects go you would be hard-pressed to find a better example of how co-production should be done.*

**User 5: Contribution**

*I very much felt included and the size of the group was ideal as many more would have lost the intimacy, and on zoom it does take a long time for everyone to have their say. So in a larger group some voices might not have been heard. The organisers were very friendly, and email contact between meetings added to cohesion. I have been involved with several short and long-term PPI- type schemes over the years. Although often quite interesting, I have usually felt, at the end, that our involvement and impact was minimal – we were there to tick a box and meet a criteria of the funding body and any positive outcomes from the involvement were not shared. P-STEP has been quite different – it was truly the meeting of different worlds – the knowledge and skills of the, usually quite young, technicians meeting the personal life experiences of the, usually older individuals living with long-term health conditions. I sincerely believe both sides learnt from each other, and the facilitators led the meetings with empathy and skill. They ensured that all participants were treated with respect and this gave the everyone confidence to air their views. The final design was better than I anticipated and it was heartening to see some of the adaptations that we had suggested had been incorporated. Compared to many past experiences, we could see the concrete evidence of our work*

**User 6: Contribution**

*Before the first video session, I felt a great deal of interest and intrigue about the focus and purpose of the sessions, wondering what and how I could contribute. There was also a sense of anticipation because, if I could actually be of help, it might be rewarding and possibly exciting. While I generally enjoy helping out with community projects, this venture was going to be something different as it was to be technical and conducted on a video link, I would be participating with other people listening in and might require particular technical or electronic skills.*

*Because of the anticipation and questions the very first session was an important moment: who would the other participants be, their age and background? These were all to be instantly overcome, however, the welcoming voice of the leader (facilitator) greeted us in such a warm and enthusiastic way that I think most of us were instantly put at ease. The initial conversation was generally chatty while each participant appeared on the screen. Each person was introduced individually by name. There were many smiles as each of us attempted to press the right buttons in order to be seen and heard with many laughs!*

*Looking back, I realise how important the first session, and the facilitator, were in reassuring everyone and setting the atmosphere for the sessions to come. We became almost a group of friends. I soon enjoyed listening to other members’ views and found it interesting to learn how they saw some things differently according to their health background and circumstances. This helped us realise that everyone’s views could be different, that we each had something valuable and unique to contribute. It certainly made me feel more than just included, it made me feel that my own contributions could actually be valuable when it came to thinking about how the app would work,*

*Once we began engaging in the design of the app these feelings about contributing became important. The facilitator gave practical examples of what might and might not work for us when we chose the route of a walk. We took it at a steady pace which allowed plenty of time to think about different situations we might meet. When you’ve never been asked such questions you don’t have ready answers and need time to think about it. For example: ‘what’s important to you when you choose a walking route?’. You need time to visualise places you normally visit and think about how you decide on where to go.*

*In each session different participants contributed first, reflecting their personal concerns and circumstances. Other members quickly became engaged, adding points about respiratory or mobility which were different but valuable. For example, I became engaged when thinking about where I’d be walking, if it’s flat, on a hill and what the gradient is and where on the app information on pollution could be found. In the process we started to think about the needs of others with different concerns who might use the app in future but were not in our group, we wanted to represent them.*

*It was an exciting moment when we first saw the app screen! It made it real; it suddenly felt a responsibility. To think we’d actively contribute to decisions about content, layout and design was interesting and also daunting. Once again our facilitator gave practical examples, gradually introducing us to members of the technical team. Each week had a different focus, e.g. size and colour of font, icons or small illustrations to make the screen user-friendly and to make sure it was acceptable to different genders, ages and user groups. Once again the mix of participants was invaluable in coming up with a range of views and new ideas.*

*During the weekly video sessions, the technicians shared their screens so we could see the options. I was surprised how worried I felt about offending the technicians – they may have spent ages designing something we were not keen on. The facilitator often stepped in and made it impersonal, such as: “this version might need a bit of tweaking..” or “we’ll have another think about this page”.*

*In our first in-person workshop It was great to put people to voices. The facilitator greeted us then had to be everywhere with so many participants and staff, to look after. I couldn’t tell who were staff and who were participants, Technical staff were helpful and knowledgeable, though seemed quieter and more serious while the others seemed more familiar with mixing with participants, joining in with the everyday chat and jokes shared amongst us ‘friends’.*

*Some technicians used technical terms we weren’t clear about or occasionally gave instructions too fast for us to follow. This became easier during the workshops when we sat looking at screens together and tried things out. We could explain our circumstances, demonstrate any difficulties and talk more openly when face to face.*

*This may always be an issue when it comes to experts working with laypeople or those not very technically savvy, but in our case we all kept going to make sure the app would work well!*

*This was my first involvement with P-STEP and on video but I have worked with similar groups in person. In some ways it was similar, i.e. that the personality and role of the facilitator was really important in setting the tone of meetings and as a role model for how members related to each other. I wonder how suitable facilitator applicants are found and appointed as they need great personal skills as well as academic knowledge. The participants had a range of backgrounds, one had a knowledge of statistics and they were from different age and ethnic groups so this made the feedback really varied. The workshop lunch in a University garden venue was unexpected and the time to mix and chat was a great opportunity. Another surprise was the support from academics in the university. It showed appreciation of users’ participation that the Professor came to meet the group, and when he spoke about the project and the app’s use it showed respect for the team.*

*Discussions on-line might be one step removed and have taken a bit longer, but actually seeing and listening to people on video was important. The design process seemed efficient time-wise, as nobody needed to travel and time-keeping was good, everything completed within an hour.*

*I wouldn’t have had the time to travel and attend in-person sessions regularly. Listening to the circumstances of those who sometimes find walking challenging gave me a great deal of insight. I gathered some may not have been able to join in regularly if it had been an in-person group so their invaluable contributions would have been missed. Travel costs were repaid and this may also have been important when deciding whether to participate –even though no one mentioned it.*

*I had never been involved in the development of an app so I didn’t know how the design process would work by involving participants. I had not used an activity app of this kind so didn’t know what to expect of it or how I would be able to interact with it and use it. Would someone ask me questions to monitor my walking? Would it actively help and guide me? Would it involve mainly text, be in colour or have pictures? I was fascinated but open-minded.*

*What was a real pleasure was experiencing how we were asked what we need, what would help us and asked to give our choices on what would be most effective. Having a second page or list was a really useful option, so we could get access to more detail if we needed it; this was helpful as we could avoid giving too much information if we didn’t want to. Choice about the size and colour of print, icons or charts was great. Not everyone can easily read small font size, as visual limitations could make reading some screens almost impossible.*

*The end result was a surprise and pleasure, even though we’d been involved all along the way. Many of the pages and decisions I’d forgotten about over the months of progress and decision-making. If you’re not usually involved in projects like this, the end result might always be a surprise and this was a good one! Access to environmental information and local features is wonderful allowing us to make choices and plans that are relevant to the day or following day. For those who sometimes or always have physical challenges it makes taking a walk more a spontaneous choice without worrying about conditions; having an app that is also attractive, ‘friendly’ and inviting almost encourages you to step out! It can ‘encourage’ and ‘congratulate’ the user - which is amazing, it’s almost like your friend!*

*In the P-STEP project users’ respiratory or mobility issues were directly relevant to the app’s design and use. Meeting online was efficient which was important in its success as nobody needed to travel, therefore time-keeping was excellent. Participants also considered the wider needs of the community if using the app, thereby representing other users whose unique contributions would otherwise have been missed. This included working with technicians and experts to make sure the design would be acceptable to different gender, age and community groups.*

*I hadn’t imagined anything like this app. It was the result of participants, leaders, experts, academics and technicians working together, bouncing new ideas off each other, solving problems and creating solutions resulting in a great design. A big thank-you to my fellow participants, the facilitator, the whole team and the University’s support. I’m very excited about using and recommending our new app!*

**User 7: Contribution**

*I have always had an interest in healthcare research and for a number of years have been involved in local patient and public involvement (PPI) initiatives within my local healthcare and university institutions.*

*Early in 2022, I accepted an invitation from a research coordinator to participate as a PPI member in a new research collaboration. This initiative involved the creation of a walking app aimed at people with health conditions which are impacted by air pollution levels. The design of the app would include the user’s ability to see information about air pollution on a planned walking route in real-time. This sounded like an interesting and worthwhile project for me to participate in as it was relevant to an existing personal health condition, as well as air quality being a current topic of concern to the general public.*

*It was also different from previous initiatives I had been involved in, as it was suggested PPI input would be needed over a longer period of time than the 1-2 meetings I had formerly experienced. In this instance we were invited to meet every three weeks and dates for a predicted collaboration period of approximately 9 months were provided well in advance. The collaboration also seemed it would be convenient to me as it was to be mainly conducted online over the Teams app, so I could remain at home and would not have to expend a large portion of time commuting or finding car parking close to a busy venue.*

*Clear and detailed information about how the Teams platform worked were supplied for those of us new to this type of interaction, which was reassuring as this technology was indeed new to me. Volunteer participants were also made aware that there may be one or possibly two face-to-face sessions later in the year, where we could meet each other along with some members of the steering team. Having this information from the beginning allowed me to see the duration of the project without feeling any pressure to attend every session, while making it possible for me to plan to contribute for the majority of the project, as I was able to fit the sessions in around other personal commitments. Another unusual strategy was that we would have an appointed PPI lead from the research team who would act as a permanent liaison for the group, acting as a single point of contact person. The planning appeared well thought out and comprehensive which further encouraged me to think I wanted to sign up for the duration of the research.*

*The first Meeting was in May 2022, and it was immediately evident that the PPI liaison team lead was taking a different approach to the collaboration from those I had previously experienced. The P-STEP team were exceptionally welcoming, warm and friendly and they facilitated introductions to the other PPI participants in a relaxed way. Having initially felt a little nervous, I was soon made to feel at ease due to the way the facilitator used their communication skills to draw us together as a group. The meeting ground rules were set and emphasised at each meeting, and included confidentiality, freedom to both express thoughts and ideas, and respect for other participant’s viewpoints. This made the forum feel like a completely safe space for sharing personal perspectives.*

*My overall impression was that a lot of thought and preparation had gone into the PPI partnership sessions. I found they were always very well resourced and organised and although the focus and topic changed for each meeting, it was always clearly outlined how they thought the contribution of the PPI was a key component of the research and that our input was genuinely going to be valued. Right from the start it was evident that the research team felt that they wanted this to be a true partnership between the researchers and the public contributors with no hint of tokenism. This was very different from other events I had participated in and it was truly refreshing.*

*Each session was planned out and dialogue, research questions and discussion accompanied by high quality resources including screen shots and videos. Often we were requested to comment on and choose between different proposed functions, screen setups, images, fonts etc which was very interesting and also resulted in some lively group debates! In between meetings personalised e-mails were sent which often included a reprise of the session, copies of information or screenshots, and also reminders for the next sessions. This meant there was a regular ongoing dialogue taking place between the team and PPI members, and insights that occurred after the session ended could still be expressed. This was another factor which led me to conclude that this project differed from others that I had been involved with in a more positive way, as in previous initiatives communication from the researchers had been more limited or generally absent altogether.*

*As the project progressed my impression that this particular collaboration was distinct from most others I had been involved with became more firmly established. Respect for the opinions of others was emphasised at each meeting, and formed the basis of my own freedom to voice my thoughts honestly, yet in a safe and supportive environment. I felt that all individual contributions, opinions and comments were clearly acknowledged, valued and most importantly we could see that our input was actually being used to develop the app as we went along. As mentioned earlier, the team were very communicative and provided regular extensive feedback about the progress of the app development and how our contributions were being incorporated. At each session, we were advised regarding the progress of the app, and this included screenshots and video presentations so we could clearly see how the elements of the app were developing and being amended as a result of our input.*

*The use of teams and the other types of technology used to bring us up to date during the sessions in this way was remarkable and it was evident within the dialogue that the team really valued the contributions we were making. The majority of the meetings included different members of the app design team working in areas directly related to the agenda for that particular session, so over time I felt I got to know some of the wider team and have an appreciation of the individuals involved and the different roles operating within the development team. This made me feel like we were part of the team, not just an external assistant.*

*Technological engagement was an important aspect of the P-STEP app development as this was an area I was largely unfamiliar with. This aspect was key to the project being an inclusive one for me, as many of the participants were also seniors, and although we were reasonably computer literate, some of us were nevertheless anxious in regard to the use of technological systems in what we all recognised was a fast-paced and constantly evolving type of environment. The thought of contributing to an app development was initially daunting, however in retrospect I realised that this was an unjustified concern. The teams IT and development designers and experts ensured that the language and concepts used remained accessible to the PPI group, and the developers and technicians were very supportive, particularly during the two face-to-face sessions.*

*During these, we were able to meet the team and each other in person, while having fun seeing the progress of the app content and experience testing some elements of the app for ourselves. The aspects we were asked to comment on or provide input for were very straightforward and although as expected opinions did vary, usually there was usually an acknowledged majority consensus by the end of the discussion. I was particularly impressed by how the team listened to individuals who raised specific issues that were relevant for them that had not occurred to the rest of us yet were significant for ensuring the app would be accessible and practicable once made publicly available. It was also evident that our comments were being recorded for the team and those not attending a specific session so they could be discussed and evaluated at a later stage.*

*The PPI group contribution to the project has now come to an end; however, we have been assured that we will be sent updates and advised that it is possible there may be one or two further contacts should any additional discussions arise as the app is refined and the final version realised. Again this indicates that we are an integral part of the team and are still seen as contributors by the researchers.*

*I am delighted that I was able to have this opportunity to volunteer for this initiative and feel that the version of the app developed to date was far better than I had imagined back at the beginning of the PPI involvement. I am confident that user contributions have made a difference in the final app content and appearance and feel my time as a volunteer within the group has been well spent. Along with the other PPI contributors, I feel proud of what we and the development team have achieved together. As mentioned, throughout my involvement I was always made to feel very much part of the team and although a PIP volunteer, my experience was that the group was a fully acknowledged and incorporated part of the research team, from the beginning and throughout the entire period of involvement.*

*On final reflection, I believe that having a main lead for the PPI liaison for the duration of the collaboration was a crucial factor in building team spirit and relationships and facilitating the success of the public/developer alliance. Throughout the meetings, this ensured that no one individual dominated, while also encouraging quieter individuals to voice their opinions. The meetings were conducted in a way which enabled all of us to feel heard and valued, and the planning, organisation and content of the sessions was excellent. I feel the continuity from having a lead individual was the key factor resulting in myself and other group members feeling this had been a very positive and enjoyable initiative to be part of. This is the first time I have been involved in a PPI initiative that has been managed in such a pro-active and comprehensive way and feel it is an excellent model for how patient involvement collaborations can add real value to research projects aimed at improving healthcare for everyone.*
