# Supplementary material for: Putting FUN into involvement: feedback user needs in the design of a mobile phone app for people with long-term conditions
Source: Res Involv Engagem. 2026 Jan 24;12:15. doi: 10.1186/s40900-026-00837-0 (PMC12870314; doi:10.1186/s40900-026-00837-0)
Supplement: Supplementary file 5 — Supplementary Material 5 [file 40900_2026_837_MOESM5_ESM.docx]

**Additional File 5: GRIPP2 Short Form**

| **Section and Topic** | **Item** |
| --- | --- |
| **1: Aim**  Report the aim of PPI in the study | P-STEP is a project designing a new exercise mobile phone app for people with long-term conditions. For P-STEP, a challenge was to make the app acceptable and usable for the users for whom it was intended. We used the term users rather than PPI in our discussions to refer to those involved in the design of the app. Barriers exist to accessing and using digital technologies, often those groups like older people with long-term conditions who may benefit the most from these types of health interventions. The attrition of using these health interventions is higher amongst these groups, and so there is a greater need to understand their digital skills, motivation, and anxieties in using these technologies. |
| **2: Methods**  Provide a clear description of the methods used for PPI in the study | We established a regular ‘core’ group of users and met every three weeks to fit with the iteration cycle of the design of the app and to enable opportunities for the design teams to interact with them. The planning of involvement activities was a collaboration between the different design teams involved in P-STEP. We called this the Feedback User Needs or FUN approach. User involvement was categorised into two phases: (i) design; (ii) validation. In the design phase, we did online discussion groups. The discussion groups were run for one hour, where users were provided with a presentation, visualisations of wireframes and discussed questions for each session to provide feedback on their needs. The online discussion groups were recorded and transcribed, supporting the writing of a feedback document of users' comments for the design teams to consider from each session. For the validation phase, two face-to-face participatory workshops were run with users to trial the P-STEP prototypes and test if they met their needs. Users were observed using the app, and their experiences were documented using workbooks and questionaries relating to their experiences. This feedback contributed to the further development of the app. |
| **3: Study results Outcomes**  Report the results of PPI in the study, including both  positive and negative outcomes | User involvement was vital for the design of the app and ensuring the suitability of project outputs, e.g., that app visualisations met user needs. The discussion sessions and workshops enable those who attended to learn from each other, both PPI members and the design teams. The design teams attended the discussion groups and workshop, enabling users to meet them and share their experiences. The design teams could observe users interacting with the early prototypes. Users were able to influence the design through this process, and they felt their involvement was positive and a meaningful experience, having an impact on the design of the app. The influence of users on the design included ensuring the ‘Home’ screen of the app was simple and accessible and that any messaging was appropriate and motivating for people with long-term conditions. Users did not report any negative outcomes and felt they were respected and listened to. |
| **4: Discussion and conclusions**  Outcomes—Comment on the extent to which PPI influenced the study overall. Describe positive and negative effects | Creating the right environment to hold conversations and including all those who wanted to participate was an important aspect of the involvement, and having adequate resources to manage interactions supported the inclusive experience in P-STEP. Good facilitation of discussions and well-organised involvement activities with the design teams worked well. The project collaborated with users mutually and productively. Good communication helped users feel valued, respected, motivated and confident. Users highlighted the importance of getting good feedback and understanding how their involvement shaped decisions about the design of P-STEP. This feedback loop was underpinned by a learning and reflexive culture within P-STEP. |
| **5: Reflections/critical perspective**  Comment critically on the study, reflecting on the things that went well  and those that did not, so others can learn from this experience | Users volunteered to contribute to the writing of this article and were asked to critically reflect on their involvement.  The reflections included were overwhelmingly positive. There may have been some anxiety about criticising the work of the involvement and design teams, given that a good relationship had been built up during the project with the teams and those involved. Potential challenges for the project included reconciling different perspectives. Tensions existed in developers wanting to create dynamic app screens and visualisations rather than basic ones, often preferred by users. Users were able to influence the designers and highlight the challenges and barriers faced by people living with long-term conditions by using technology interventions like mobile phone apps. There was a risk that not involving users who may find technology difficult in a meaningful way would only see validation of what was being delivered and designed by developers, rather than challenging their designs. Having an inclusive and democratic way of working with users, however, should ensure their voices are heard in these types of projects, and they can influence design teams and increase the chances that the end product will meet users’ needs and be successful. |
